# Interpreting plural predication: homogeneity and non-maximality

**DOI:** 10.1007/s10988-020-09311-w

**Published:** 2020-11-03

**Authors:** Manuel Križ, Benjamin Spector

**Affiliations:** 1 Department of Linguistics, University of Vienna, Wien, Austria; 2 Institut Jean Nicod, Département d’études cognitives, ENS, CNRS, EHESS, PSL Research University, CNRS, Paris, France

**Keywords:** Pluralities, Homogeneity, Non-maximality, Imprecision, Definite descriptions

## Abstract

Plural definite descriptions across many languages display two well-known properties. First, they can give rise to so-called non-maximal readings, in the sense that they ‘allow for exceptions’ (*Mary read the books on the reading list*, in some contexts, can be judged true even if Mary didn’t read all the books on the reading list). Second, while they tend to have a quasi-universal quantificational force in affirmative sentences (‘quasi-universal’ rather than simply ‘universal’ due to the possibility of exceptions we have just mentioned), they tend to be interpreted existentially in the scope of negation (a property often referred to as *homogeneity*, cf. Löbner in Linguist Philos 23:213–308, 2000). Building on previous works (in particular Krifka in Proceedings of SALT VI, Cornell University, pp 136–153, 1996 and Malamud in Semant Pragmat, 5:1–28, 2012), we offer a theory in which sentences containing plural definite expressions trigger a family of possible interpretations, and where general principles of language use account for their interpretation in various contexts and syntactic environments. Our theory solves a number of problems that these previous works encounter, and has broader empirical coverage in that it offers a precise analysis for sentences that display complex interactions between plural definites, quantifiers and bound variables, as well as for cases involving non-distributive predicates. The resulting proposal is briefly compared with an alternative proposal by Križ (Aspects of homogeneity in the semantics of natural language, University of Vienna, 2015), which has similar coverage but is based on a very different architecture and sometimes makes subtly different predictions.

## Introduction

Plural definite descriptions across many languages display two well-known properties. First, they can give rise to so-called non-maximal readings, in the sense that they ‘allow for exceptions’. That is, the sentence in (1), in some contexts, can be judged true even if there are a few of the relevant books that Mary didn’t read. Second, while they give rise to a quasi-universal interpretation in affirmative sentences (‘quasi-universal’ rather than simply ‘universal’ due to the possibility of exceptions that we have just mentioned), they tend to be interpreted existentially in the scope of negation, as illustrated in (2)—a phenomenon that is known in the literature as *homogeneity*. (2b) does not have truth conditions that are complementary to those of (2a), that is to say, it does not simply mean that Mary didn’t read all of the books on the reading list. Rather, it conveys that Mary read no book, or nearly no book, on the reading list. 
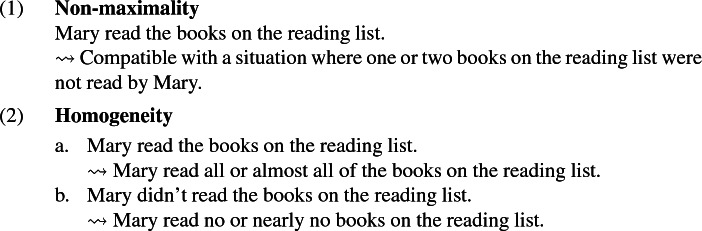
 There are reasons to think that these two properties (homogeneity and non-maximality) are the two sides of the same coin. This has recently been suggested by Malamud (2012), and argued for in greater detail by Križ (2016). The main argument is that the two phenomena appear (and disappear) together: the addition of *all* removes both non-maximality and homogeneity. Regarding non-maximality, this is the so-called slack-regulating effect of *all* that has been recognised by Brisson (1998), Lasersohn (1999), and Burnett (2012).^1^ Suppose that Mary has a list of books she should read to prepare for an exam, but one of these books in fact wouldn’t do much to help her chances at the exam. If she reads all the books on her list except this one, it would be fine to report this situation by using (3). (4), however, would be considered false.
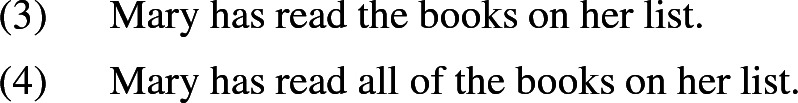
 Homogeneity, too, is removed by *all* (Löbner 2000). That is to say, the negation of (4) does not imply that Mary has read *no* books on her list. Rather, (5) is true as soon as there is one book on her list that Mary hasn’t read.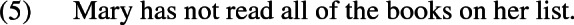 These facts raise a number of issues that this paper aims to address. At the conceptual level, we would like to derive these two properties (homogeneity and non-maximality) from (i) an adequate semantics for sentences that contain definite descriptions, and (ii) general principles of language use. At the empirical level, we would like to know under which conditions non-maximal readings arise, on the one hand, and, on the other hand, how the existential vs. quasi-universal apparent ambiguity is resolved in more complex syntactic contexts. The goal of this paper is to provide a theory that addresses these two questions, and to provide an account that also explains why these properties are jointly targeted by *all*.

In a nutshell, we will argue that both homogeneity and non-maximality are the by-products of a more fundamental fact: the truth-conditions for sentences in which a predicate is applied to expressions that denote complex objects are *underspecified*. Of the two proposals that we are aware of that attempt to link homogeneity and non-maximality, Malamud’s (2012) runs into serious problems and is of narrower scope in that it does not address the interactions between plurals, definites, quantification and pronouns. Križ (2015, (2016) offers a theory that covers most of this ground, but has a radically different architecture. In this paper, we offer a theory that is much closer in spirit to Malamud’s and makes substantive predictions for a wide range of cases, sometimes subtly different from those of Križ’s (2016). Thus, our account can be viewed as a way to fix problems and overcome limitations of some of the previous literature.

We will associate with sentences such as the ones in (1) and (2) a *family of interpretations*, which we will call *candidate interpretations*. Some of these candidates will be filtered out by considerations of relevance (following Malamud’s (2012) insights). Most often, however, several candidates will pass this test, and we will propose a procedure which, given a set of remaining candidates, assigns an overall interpretation to the sentence. This procedure will be responsible for homogeneity effects. While the general architecture of our system is quite close to Malamud’s, our proposal differs from hers both in terms of its empirical predictions and its conceptual motivations. Furthermore, contrary to most of the previous works, we provide an explicit recursive semantics that generates the input of the application of pragmatic principles that allows us to deal adequately with sentences containing bound plural pronouns and co-referential expressions.

The paper is structured in two parts. Part one consists of Sects. 2–4. In this part, we lay out how we take inspiration from the literature, and how we amend these proposals to obtain our final theory. In particular, we are concerned with two questions: first, we discuss between which propositions, precisely, the meaning of a sentence with plural predication is underspecified. Second, we discuss the pragmatic rules that govern the use of such underspecified sentences. Part one of the paper can be read in isolation.

Part two of the paper, which begins in Sect. 5 and presupposes part one, is then concerned with the problem of how to compositionally derive the candidate interpretations of a sentence while predicting the right behaviour for complex sentences with negation, coordination, and quantification.

## Semantic underdetermination: previous accounts

Our account belongs to a class of theories in which the semantic meaning of definite plurals (or, more accurately, sentences that contain them) is somehow underspecified and where some pragmatic principle plays a role in choosing between different possible meanings (Krifka 1996; Lasersohn 1999; Winter 2001; Malamud 2012, and, in a way, Brisson 1998; see Dalrymple et al. 1994, 1998; Sabato and Winter 2012 for the same in connection with reciprocals.). Two previous works, Krifka (1996) and Malamud (2012), are particularly relevant to our own theory, which in some respects is inspired by them.^2^ We will argue, however, that both proposals face problems that are resolved by our own. Krifka’s is, mostly, a theory of homogeneity, but we will show that it does not correctly predict the way homogeneity inferences project, before suggesting an amendment to solve this problem. At this point, however, we will still have no story for non-maximality. This is where Malamud’s proposal comes in. We will see that it, too, encounters serious problems, but that we can borrow some of its ingredients to reach an empirically adequate theory of both homogeneity and non-maximality.

### From the SMH to truth on all readings

Krifka’s (1996) account of homogeneity is the following: a plural definite is underspecified between an existential and a universal interpretation, and the strongest denotation for the sentence is preferred as long as it is consistent with general background assumptions. This principle is known as the *Strongest Meaning Hypothesis* (SMH), a term coined by Dalrymple et al. (1994). It follows that a definite plural is interpreted universally in an upward-entailing context and existentially in a downward-entailing one. Thus, the sentence in (6) has the two candidate interpretations in (6a) and (6b), and (6b), being logically stronger, is chosen. Conversely, in (7), (7a) is stronger.^3^
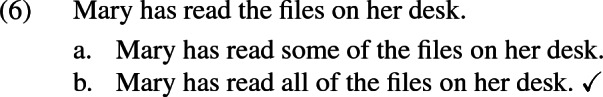

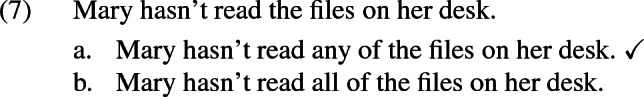


If one takes the universal interpretation not to involve a universal quantifier over individuals in the strict logical sense, but rather to be akin to *all*, then this immediately predicts homogeneity also for collective predicates, since the entailment from *all* to *some* is not restricted to distributive predicates. As we will see, this is an important virtue of Krifka’s approach that needs to be preserved.
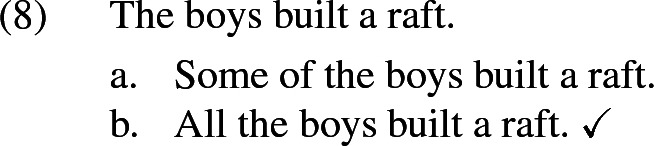

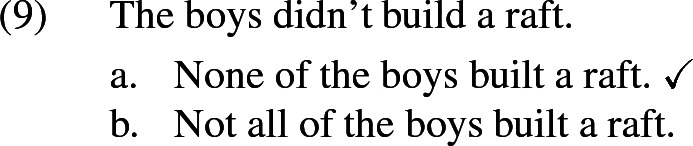


The use of the SMH to capture the phenomena is problematic, however, as it fails yield a satisfactory result when the definite plural occurs in a non-monotonic context (Spector 2013; Magri 2014), as in (10). The most natural reading of (10) is that exactly one student read all of the books and all of the others read none (see Križ and Emmanuel 2015 for experimental data supporting this claim), i.e. the *conjunction* of the two candidates in (10a) and (10b). 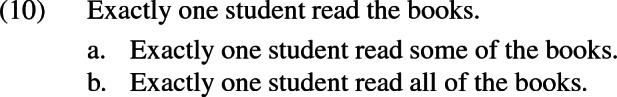


There is, however, an easy fix based on a slight shift in perspective. When trying to determine the meaning of a semantically underdetermined sentence, we don’t choose the *strongest* of the ‘candidate interpretations’, but we take their *conjunction*. The idea is that a sentence is considered *true* if all its candidate interpretations are true, false if all of them are false, neither true nor false otherwise. Now, for simple cases, based on Krifka’s candidate interpretations, we have only two candidate interpretations. And whenever the plural definite occurs in a monotonic context, one of them entails the other, so that the conjunction of both readings is equivalent to the stronger reading. We thus make the same predictions as Krifka whenever a plural definite occurs in a monotonic context. But we do better for (10), since (10) is now predicted to be perceived as true if and only if there is one student who read all the books and all other students read no books, which we argued is the most natural understanding of the sentence.

Since, based on this procedure, a sentence can fail to be assigned a truth value (namely when some but not all of the candidate interpretations are true), we have defined a kind of *trivalent semantics*, which is in line with Löbner’s (2000) approach.^4^ We do not explore this in detail, but in Sect. 3.5 we suggest and discuss a pragmatic motivation for why a divergence between the different candidate interpretations may be treated as a failure of the sentence to have a determinate truth value overall.

### Non-maximality and candidate interpretations: Malamud (2012)

So far, non-maximality isn’t part of the picture, but an avenue is in sight for importing it. One could make the candidate interpretations more fine-grained and have a pragmatic mechanism filter them, so that sometimes not *all* of them have to be true for the sentence to be judged true.

A somewhat related idea has been worked out by Malamud (2012). While we will draw inspiration from her proposal, it turns out that it runs into quite serious problems. In this section, we first present the essential ingredients of Malamud’s story, and then explain what these problems are.

#### Malamud’s proposal

Semi-formally speaking, Malamud (2012) associates a sentence in which a predicate is applied to a plurality *X* with a set of propositions that speak about all mereological parts of *X* (not necessarily atomic ones). Thus, if the sentence in question is “X are P”, then the set of propositions in question is

$$\begin{aligned} \{P(Y)|Y\sqsubseteq X\}. \end{aligned}$$

^5^

For example, if there are three^6^ books *a*, *b*, and *c*, then the sentence in (11a) is associated with the set of propositions in (11b). 
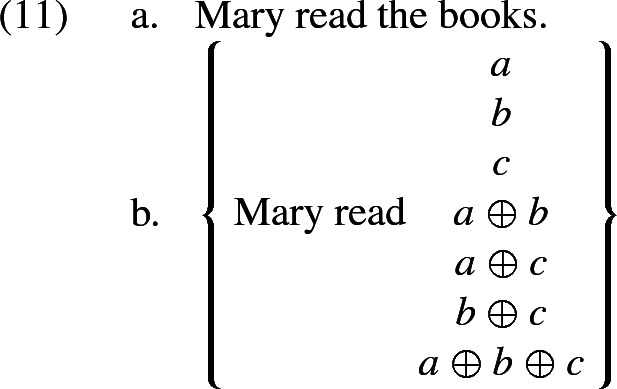


To obtain the final meaning, we take only those propositions that are maximally relevant for current purposes and then form their disjunction. The notion of ‘maximal relevance’ used by Malamud is fairly complex in the general case. However, given the additional assumptions she makes, it boils down in practice to the following. We posit that ‘current purposes’ are appropriately modelled by a partition of the set of possible worlds, and that the addressee’s only goal is to know in which cell of the partition she is. Then $\phi$ is ‘more relevant than’ $\psi$ if $\phi$ eliminates more equivalence classes than $\psi$ does.

And for $\phi$ to count as maximally relevant in a set of propositions $\Sigma$, it should simply be the case that there is no $\psi$ in $\Sigma$, distinct from $\phi$, such that $\psi$ is more relevant than $\phi$. Note that this notion of maximality is weak, in that several candidates can count as maximally relevant—a fact that will turn out to be crucial later on.

Let us now illustrate how this works for a simple sentence such as (11a). First let us consider the maximally fine-grained partition corresponding to the case where the addressee wants to know as much as possible about which books Mary read. In other words, each cell of the partition is characterised by which books Mary read. It is clear, in this case, that there is only one maximally relevant candidate interpretation, namely *Mary read* ${a \oplus b \oplus c}$. Indeed, this candidate is compatible with only one cell, the one where Mary read ${a \oplus b \oplus c}$, while all other candidates are compatible with that very cell and also at least one other. So, in this case, the set of ‘maximally relevant propositions’ is the singleton set whose only member is *Mary read* ${a \oplus b \oplus c}$. That proposition therefore comes out as the overall interpretation of the sentence.

Consider now another case, where what matters is (i) whether Mary read at least two of the three books, and (ii) whether she read any books at all. Then we have three cells: $i_1$ where she read two or three books, $i_2$ where she read one, and $i_3$ where she didn’t read any. Now we look at the alternative propositions. Those about a single book eliminate one cell: $i_3$. The others eliminate two cells, $i_3$ and $i_2$, thereby uniquely determining $i_1$. Those that eliminate two cells are the maximally relevant candidates. Thus, to obtain the final meaning, we form the disjunction of all the propositions that eliminate two cells, obtaining (12). 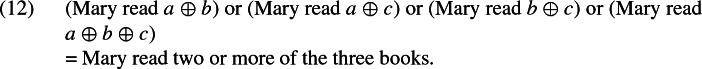


This captures Malamud’s insight that non-maximal readings arise when ‘exceptions’ are in a certain sense not relevant, and that a given sentence can receive quite a number of different non-maximal construals, depending on what is taken to be relevant.

A version of the example closer to life might look something like this. Assume that Mary has a list of books, but some of the books on it would not help her chances at passing an exam. Then, uttered in the context of how well-prepared Mary is, (13) might easily be judged true as long as the books she has read are enough to ensure that she will pass. 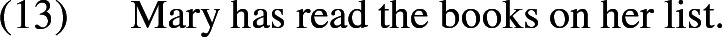


In fact, it seems that non-maximality can go so far as to yield basically existential readings. Malamud (2012) discusses the following scenario.

**Situation:** Mary has a large house with over a dozen windows in different rooms. She locks up and leaves to go on a road trip with her friend Max, forgetting to close just a few of the many windows in various rooms. A few minutes into the ride, Max says, “There is a thunderstorm coming. Is the house going to be OK?” Mary replies:

${}^\textsc {ok}$**Utterance:** Oh my, we have to go back—the windows are open!

${}^\textsc {ok}$**Utterance:** Oh my, we have to go back—the windows aren’t closed!

Malamud’s procedure, as described above, predicts these facts, and this is a virtue of her proposal that we will integrate in our own. However, her theory runs into three problems (Križ 2016). The most serious one is that her account actually fails to predict the homogeneity property, i.e. the correct interpretation of sentences where a plural definite is in the scope of negation—despite her claim to the contrary. A second problem, though, is that Malamud’s account inherits the problems faced by Krifka’s: it makes incorrect predictions for cases where a plural definite occurs in a non-monotonic context. A third problem involves sentences where a plural definite is the argument of a collective predicate.

#### Problem #1: homogeneity

In a context where everything is relevant, the partition relative to which maximal relevance is assessed is the maximally fine-grained partition, where each world is its own equivalence class. In such a context, maximal relevance is most often equivalent to maximal logical strength. For *Mary read the books*, the maximally relevant candidate interpretation is the proposition that Mary read all the books, which entails all other candidate meanings. For this reason, Malamud suggests that her system can make the same prediction as a theory based on the SMH (such as Krifka’s), the idea being that choosing the maximally relevant candidates would amount, in a lot of contexts, to choosing the most informative candidate. However, this conclusion is mistaken in the case of negative sentences, as noted in Križ (2016). We show here why this is the case, and then discuss a revised version of Malamud’s proposal that solves this particular problem and brings us somewhat closer to our own eventual theory.

Consider the negative sentence (14a) and its associated set of candidate interpretations in (14b).
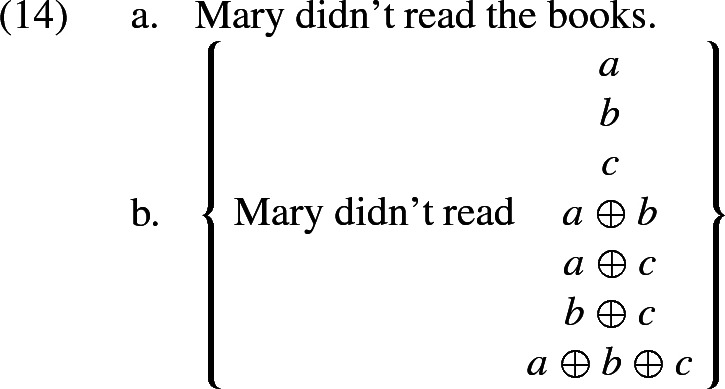


Assume again that the underlying partition is maximally fine grained. We furthermore take it (as Malamud does) that the underlying logic is bivalent, and that the proposition informally represented above by *Mary didn’t read* ${a\oplus b}$ is true as soon as she didn’t read both *a* and *b*. Then the most informative candidates (hence the maximally relevant ones) are the three propositions *Mary didn’t read a*, *Mary didn’t read b*, *Mary didn’t read c*. In Malamud’s account, the final interpretation is the *disjunction* of the maximally relevant candidates, not the conjunction. But the disjunction of these three maximally relevant candidates is simply the proposition that Mary didn’t read all the books—and not the proposition that Mary didn’t read any books.^7^

#### Problem #2: non-monotonic contexts

Malamud’s proposal, fails to derive the right results when a plural referring expression occurs in a non-monotonic context, such as the scope of *exactly one girl*. 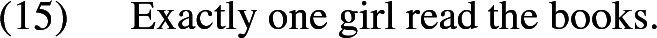 Assume, again, that there are three books *a*, *b*, and *c*, and that, for each girl, the adressee is interested in knowing which of the three books this girl read. Then (15) tends to be understood as suggesting that one of the girls read all the books and the others read none.^8^ Let us see what Malamud’s proposal, or our revised version of her proposal, delivers in such a case.

The candidates that are generated on Malamud’s theory are the following: 
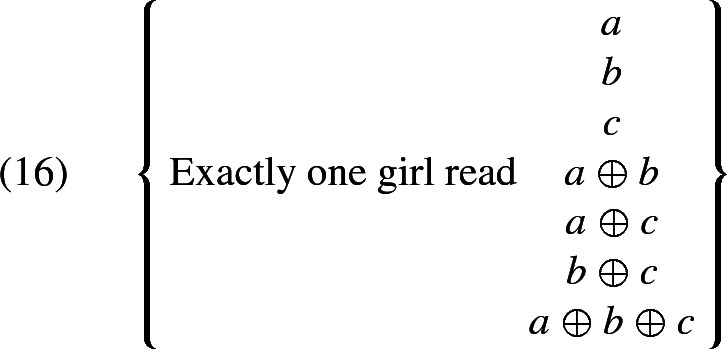
 Regardless of how exactly relevance filters out candidate propositions, we can reason as follows. The interpretation that we would like to derive entails that there is a girl who read the three books. The only candidate proposition that has this entailment is the last one, *Exactly one girl read* ${a \oplus b \oplus c}$, which we call $\phi$ for convenience. Furthermore, among the propositions that can be obtained by taking the disjunction of a subset of the candidates, only $\phi$ has this entailment (because for any candidate proposition $\psi$ distinct from $\phi$, $\psi$ does not have this entailment, and therefore $\phi \vee \psi$ doesn’t either). Therefore, the only way for Malamud’s system to generate a reading for (15) that entails that a girl read all the books would be by constructing a context where $\phi$ is the only maximally relevant candidate. But then, (15) would be understood to mean $\phi$, but $\phi$ fails to entail that no girl apart from the one who read the three books read any books. We can conclude that there is no way the interpretation we are after can be generated on Malamud’s account.

#### Collective predicates

With distributive predicates like *read*, the candidate propositions are (partially) ordered by logical strength. “Mary read ${a\oplus b}$” entails “Mary read *a*”, etc. In particular, there is always a maximally strong alternative which involves the entire plurality in question and entails all other alternatives. Problems for Malamud’s theory arise when the alternatives are logically independent, which happens with collective predicates (Križ 2016). Consider the sentence (17a), with an informal representation of the associated alternative propositions in (17b). 
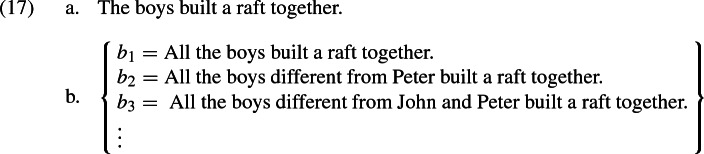


Of those alternatives, $b_1$ is maximal in the sense that it involves the maximal plurality, but it is logically independent from the other alternatives, as those are from each other. In a situation where all the boys together built a raft, $b_2$ isn’t true, as “build a raft together” doesn’t just mean “participate in building a raft”.^9^ Conversely, $b_1$ isn’t true when Peter didn’t actually participate. Hence, neither of the alternative propositions entail each other.^10^

Now assume again that the underlying partition is maximally fine-grained. That is, participants are interested in knowing, for each plurality of boys, whether that plurality built a raft together (maybe the underlying question is ‘What did every boy do today?’). Then each candidate rules out exactly the same number of cells (namely it is compatible with all cells except those in which a certain plurality of boys did not build a raft). As a result they are all maximally relevant, and the resulting interpretation, on Malamud’s account, is the grand disjunction of all the candidates, which is itself equivalent to “Some boys built a raft together”, which is overly weak. Note that if we took the grand conjunction of all candidates instead (as we will do in our own proposal, but based on a different definition of candidates), we would get a result which is much too strong, namely the proposition that for every subgroup of boys, they built a raft together!

## Semantic underdetermination: our account

The general architecture of our proposal is as follows. Like Krifka and Malamud, and also Lasersohn (1999), we associate with a plural definite a set of *candidate denotations*. From this we derive *candidate interpretations* (by interpreting a plural definite in a sentence as denoting some candidate denotation or other).^11^ Then we filter out some of the candidate propositions by relevance considerations. Finally, the sentence will count as true if all the remaining candidate propositions are true, false if all the remaining candidates are false, and undefined otherwise. In this section we do not yet offer explicit compositional rules that derive candidate propositions—this will be the topic of Sect. 5—, but we show the basic workings of our system for a number of relatively simple cases.

### The proper form of candidates

As a starting point, let us return to Krifka’s proposal: there are two candidate denotations for plural definites, one in which the definite plural is interpreted as an existential with lowest scope, and one in which it is a low-scope universal. In order to have available the finer distinctions needed to describe non-maximal readings, what we will do first is to, as it were, populate the space between the universal and the existential with further candidate denotations.

Let us notate the restrictor plurality under consideration as *x* and the set of its (not necessarily atomic) mereological parts as ${\mathcal Part}(x)$. The universal candidate denotation is, in effect, simply *x*.^12^ The existential alternative is the disjunction of all mereological parts of *x*, i.e. $\bigvee \mathcal {P}art(x)$. A simple way to add more candidates is to say that a candidate is any disjunction of mereolocial parts of the restrictor plurality, resulting in the set of candidates in (18) (in contrast with Malamud’s approach, where candidate denotations are only the mereological parts of *x*, but do not include disjunctions of such parts).
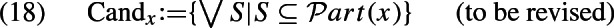


Assuming that ${x= a\oplus b\oplus c}$, some members of $\text {Cand}_x$ are exemplified below:
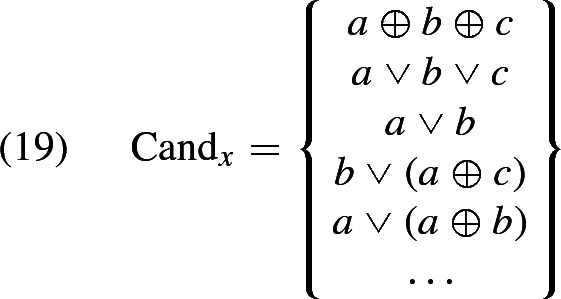
 Informally, the *candidate propositions* for a sentence of the form *S(the NPs)*, where *x* is the sum of all the members of the denotation of *NPs*, will be all the propositions that one gets by interpreting *the NPs* as denoting some member of $\text {Cand}_x$. In cases where all these candidate interpretations pass the test of relevance (which will be the case, in particular, in a context where everything counts as relevant), the final interpretation will be the conjunction of all the candidate propositions. However, this would only work for distributive predicates, for the reasons discussed in Sect. 2.2.4. The problem is that the only way to make all of these candidates true for a sentence such as *The boys built a raft together* is for the predicate to be true of all parts of *x*, i.e. the sentence will be wrongly predicted to mean that every subplurality of the boys built a raft. But what it means for a predicate to be collective is precisely that it can be true of a plurality without being true of its parts. Thus, we have to put a further restriction on the shape of our candidates. Intuitively, we want the candidates to be disjunctions that are *based* on a subset *S* of $\mathcal {P}art(x)$, but which are also true if the predicate is fulfilled by any individual that merely contains a member of *S* as a mereological part. For example, if we base a candidate proposition on ${a\oplus b}$ in the above example, we want to interpret the relevant plural definite as ${(a \oplus b) \vee (a \oplus b \oplus c)}$. When the predicate is distributive, this reduces to ${a \oplus b}$, but this is not so, in general, when the predicate is collective. If this candidate ${((a \oplus b) \vee (a \oplus b \oplus c))}$ happens to be the only relevant one (in a sense we will discuss later), the sentence *The boys built a raft together* will be interpreted as *A plurality of boys that contains* ${a \oplus b}$ *built a raft together*. So we define the set of candidates as follows:
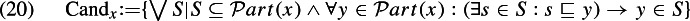


With the aid of some auxiliary notations, the following is an equivalent formulation:
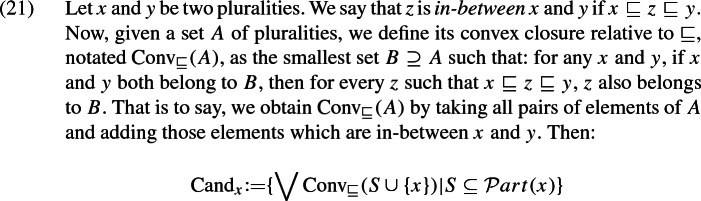


We can thus obtain a candidate derived from *x* by following these steps:

1. Pick a set *S* of parts of *x*.
2. Add *x* to that set (call the result $S'$).
3. Form the convex closure of $S'$ by adding all individuals that are mereologically in-between *x* and any of the parts we picked in step 1. Call the result $S''$.
4. Take the existential quantifier with domain $S''$.^13^^,^^14^

Now all candidates are entailed by the universal candidate, and so collective predicates make all candidates true when they hold of the maximal individual *x*.^15^

For illustration, assume that we are trying to find candidates on the basis of the plurality $j\oplus m\oplus b$, consisting of John, Mary, and Bill. We take a set of parts of this plurality, for example $\{j,m\}$. The candidate resulting from this set of parts is $j \vee m \vee j \oplus b \vee m \oplus b \vee j \oplus m \vee j \oplus m \oplus b$, approximated in words: John, or Mary, or any plurality among John, Mary and Bill that contains either John or Mary (or both). When we combine this candidate meaning with a distributive predicate such as ‘came to the party’, the resulting proposition is, informally, ‘John, or Mary, or any plurality among John, Mary and Bill that contains either John or Mary (or both) came to the party’, which reduces to ‘John or Mary came to the party’. For another candidate, we could also have started with the set $\{j, b\oplus m\}$, in which case the resulting candidate would be $j \vee j~{\oplus }~b \vee j~{\oplus }~m \vee b~{\oplus }~m \vee j~{\oplus }~m~{\oplus }~b$, i.e. John, or Mary and Bill, or any plurality among the three children containing either John, or both Mary and Bill. Combining this meaning with that of ‘came to the party’, the resulting proposition is ‘Either John, or Mary and Bill, are part of a subplurality of $j\oplus m \oplus b$ that came to the party’, which reduces to ‘John or Mary and Bill came to the party’. Generally speaking, any candidate for *The students came to the party’* amounts to *Q came to the party*, where *Q* is an increasing generalised quantifier whose smallest-live on set (its restrictor) is the set of students. Things are, however, different with collective predicates. With the candidate denotation based on $\{j, m\}$, the resulting meaning when it combines with, say, *lifted the piano*, is the proposition that states that a subplurality of $j~{\oplus }~m~{\oplus }~b$ that contains *j* or *m* or both lifted the piano, i.e. John or Mary or both contributed to a lifting of the piano, to which nobody else except maybe Bill contributed.

Putting aside for a moment the issue of non-maximality, the simplest version of our proposal says that a sentence is judged true if all the candidate propositions that correspond to this sentence are true, false if all these candidate propositions are false, undefined otherwise. For definite descriptions in monotonic contexts, we make the same predictions as with a simple two-candidate version. However, the enrichment of the set of candidates slightly changes the predictions in the case of non-monotonic contexts. Consider a scenario with three students and some number of books. Mary read all of the books, Sue read half of them, and Peter also read half of them, but Peter and Sue didn’t read the same books. On the Krifka-inspired story with just two alternatives, the sentence (22) will be false in this scenario, since both its associated candidate interpretations are false: (22a) is false because, in fact, all three students read some of the books, and (22b) is false because only one student read all of the books.
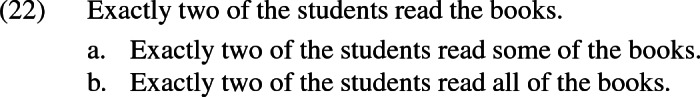


However, on our new theory we have additional candidates, some of which are true. If, for example, Peter read books *a*, *b*, and *c*, which Sue didn’t read, then the (informally represented) candidate (23) is true: Mary and Peter read *a*, *b*, and *c*, and Sue didn’t.
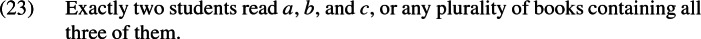


Thus, we predict (22) to be neither true nor false in the given scenario. This is a potentially problematic result, as it is at odds with the experimental findings in Križ and Emmanuel (2015). In their ternary truth-value judgement task, with options *completely true*, *completely false*, and *neither*, subjects judged *exactly*-sentences false, rather than undefined, in the crucial type of scenario. In particular, sentences of the form (24a) were judged false in scenarios of the form (24b).^16^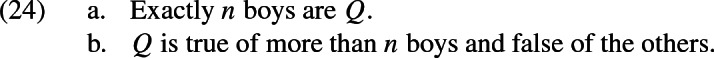 In their experimental items, *Q* contained a bound variable—it was *found their presents*—and the image presented was such that no two boys found the same present. Thus, our theory predicts undefinedness in all those cases, rather than the observed falsity.

There may, however, be a way out of this. In the next section, we will discuss in some detail how we propose to capture non-maximality: by filtering candidate meanings through a notion of relevance, excluding from consideration those that are not relevant to the conversation at hand. It is plausible that subjects would solve Križ and Chemla’s task by imagining different contexts and judging the sentence false if it is (potentially non-maximally) false in all of those contexts. The candidates that are responsible for the problematic prediction of our theory are quite complex, and so the contexts that are required to make them relevant are quite odd. It is therefore possible that subjects might simply fail to imagine these rather outlandish contexts; instead, they imagine more natural contexts in which the offending candidates are not relevant and are therefore excluded from consideration. Then, the sentence will be false in all the various contexts imagined, and subjects judge it *completely false* because they find, introspectively, that no matter what context they imagine, they would find it false.

In any event, it is clear that the truth-conditions of (22) come out correctly on our story: the sentence is predicted to be judged true (putting aside non-maximality) if and only if there are two students who read all the books, and all the others read no books.

### Capturing non-maximality

With such sets of candidate interpretations at our disposal, we need to bring non-maximality into the picture. The set of candidate interpretations should be filtered somehow based on pragmatic factors, potentially removing some candidates and thereby weakening the meaning. What could this look like, specifically? We suggest that what happens is that those candidate interpretations are thrown out that are not relevant for the current purposes of the conversation.

To make a precise theory of this, we have to operationalise the notions of current purposes and relevance. Following van Rooij (2003), Malamud (2012), and Križ (2016), who had similar or identical goals, we represent the current purposes of the conversation by a partition of the common ground, viewed as a set of possible worlds. The participants of the conversation want to know which cell the actual world is in, but for any given cell, they don’t care which particular world in that cell they are in. Reserving the expression *current purposes* for the intuitive notion, we will refer to this partition as the *current issue*.

We say that a proposition is *(strongly) relevant* to an issue (a partition) iff it eliminates at least one cell and furthermore does not contain information that doesn’t help with deciding which cell we are in. In other words, a proposition is strongly relevant relative to a partition just in case it espouses the contour of the partition: if the proposition is true in a given world *w*, then it is true in all the worlds that belong to the equivalence class of *w*. It can be formally characterised as below, where I is a partition of the set of possible worlds.
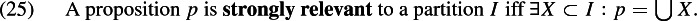


The idea is that a sentence is judged only on the basis of the strongly relevant candidate propositions. In particular, it is judged true if all the strongly relevant candidate propositions are true, and false if all of them are false.^17^ To see how this works, take a very simple toy scenario: there are ten books, and for current purposes (maybe the underlying question is whether Mary read enough books on the reading list so that she can do the exam), reading eight or nine of them is as good as reading all of them, whereas readings only one or two is as bad as reading none. Then, if we restrict our attention to the individual Mary, the current issue has three cells:
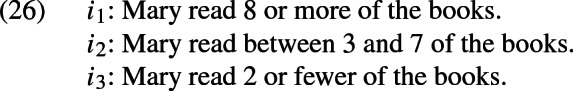


It is easy to see what happens when we now interpret the sentence in (27) with respect to this issue. The proposition $i_1$ is, in fact, one of the candidate interpretations of that sentence, and it is strongly relevant. The same holds for $i_1\cup i_2$, i.e. the proposition that Mary read three or more of the books. These two propositions are, indeed, the only strongly relevant candidate interpretations, and so the overall meaning we obtain is their conjunction: that Mary read eight or more books, i.e. $i_1$.
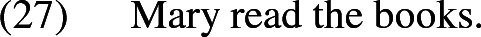


Similarly, we see non-maximality also on the negative side. The two strongly relevant candidate interpretations of (28) are exactly the negations of $i_1$ and $i_1\cup i_2$, so it ends up meaning that Mary read two or fewer of the books, which is exactly the proposition $i_3$. Note that non-maximal readings on both ends are still quite compatible with the existence of a gap: if we are in $i_2$, both (27) and (28) are neither true nor false even on their non-maximal readings.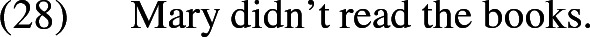 Let us now turn to the more challenging kind of example that contains a definite plural in a non-monotonic context, like (29).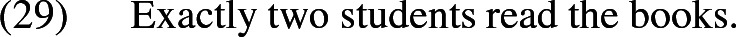 Assume again that reading eight or more of the books is as good as reading all, and that reading two or fewer is as bad as reading none, and that what we are interested in is how many students fall into which category. Let’s call the categories $C_1$, $C_2$, and $C_3$, in analogy to the cells in the issue of the previous example. Now there are again two candidate interpretations that are strongly relevant. (30a) says that exactly two students fall into $C_1$, while (30b) says that exactly two students fall into $C_1\cup C_2$.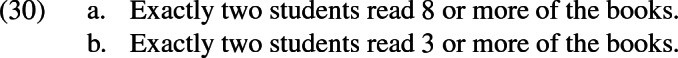 To obtain the final meaning of (29), we conjoin those two relevant candidate interpretations: exactly two students read two or more of the books, and all the others read two or fewer of the books.

### Upward homogeneity

Križ (2016) discusses the fact that collective predicates do not only give rise to standard homogeneity effects, but also to what he calls *upward homogeneity*. Consider the following pair: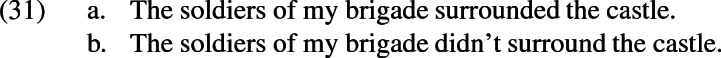 While (31a) implies that (roughly) all the soldiers of my brigade together surrounded the case, (31b) suggests that (roughly) no soldiers in my brigade participated in any surrounding of the castle. So far, the prediction we make about (31a) is that the sentence is undefined if some but not all of the soldiers of my brigade formed a circle around the castle on their own, and true if no plurality of soldiers of my brigade formed a circle around the castle together. The sentence is predicted to be true, in particular, if all the soldiers of my brigade *together with other soldiers* formed a circle around the castle.

Križ (2016) gives a unified definition of homogeneity that makes the sentence undefined in this scenario, thus capturing this new observation together with the homogeneity effects we have so far been discussing.^18^ Upward homogeneity can be captured on our approach, albeit in a somewhat stipulative way, if we modify the definition of candidate denotations. We show this in the appendix, but leave the matter aside in the discussion to follow.

### Homogeneity and downward-entailing contexts

Križ and Emmanuel (2015) found in their experiments that definite plurals which are unambiguously embedded in a downward-entailing environment receive, as we would expect, and effectively existential interpretation.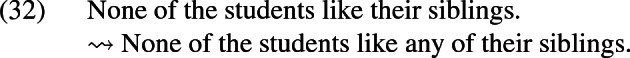 However they found that subjects mostly (though not universally) treated such sentences as simply false in a situation where some, but not all candidate denotations were true, which for (32) would mean that at least one student likes some of his siblings, but no student likes all of his siblings. This differed from all other contexts in which definite plurals were tested, where their experiment more pronouncedly showed an intermediate status for sentences where some, but not all candidate denotations were true.

Bar-Lev (2018) has taken this to motivate an implicature-based account of homogeneity: the literal meaning of a definite plural is existential, and to the extent that its meaning ends up closer to the universal, this is a strengthening implicature. A sentence with a definite plural in an upward-entailing context can thus have an intermediate status for a homogeneity violation: its literal meaning is true, but its implicature is false (whereas in our theory, that intermediate status is constituted by some candidate interpretations being true and others being false). In a downward-entailing environment, however, no implicature arises and so the sentence can never have an intermediate status. Consequently, no non-maximality is predicted to arise in such sentences, either (on both a conceptual level, since the two phenomena are inseparable, and given how non-maximality is implemented in Bar-Lev’s particular system).

We believe that this is an overreaction to the admittedly initially unexpected experimental findings. First, the experiment did not show a total absence of intermediate judgements, merely a reduced rate. Second, we think that non-maximality, while comparatively rare, is nevertheless possible with such sentences.

Consider, for example, (33), inspired by Malamud’s example discussed in Sect. 2.2.1 above.

It appears to us that non-maximality is equally possible in the universal and the negative existential statement, so that (33a) and (33b) are effectively synonymous.^19^ In both case, the sentence is true as soon as some fraction of some house’s windows are open. Crucially, (33b) does not really require every window of every house to be open, thus showing a non-maximal interpretation of *closed* with respect to the relevant plurality of windows.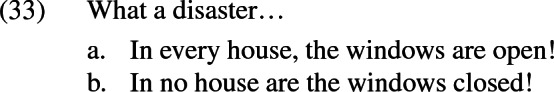 This is not very well compatible with Bar-Lev’s (2018) approach. In order to account for such a reading, he would have to posit local implicatures in the scope of a negative existential; but local implicatures in downward-entailing contexts which weaken the overall meaning of the sentence are generally taken not to exist or to be extremely marked (Chierchia et al. 2012; Fox and Spector 2018).

On our theory, however, there is no asymmetry in principle: both (33a) and (33b) are associated with a set of candidate interpretations which are filtered by context to obtain non-maximal readings and which confer an intermediate status upon the sentence when some of them are true and others false.

Of course, we need an explanation as to why non-maximal readings appear less available in negative environments. We speculate that the boundary between “none” and “some” is more cognitively salient than the boundary between “all” and “almost all”, so that there are fewer contexts in ordinary life which are indifferent to that boundary (as required to obtain a non-maximal reading). Additionally, we need an assumption about the manner in which Križ and Chemla’s (2015) performed the task: in a loose sense, they would choose an intermediate response (for a non-true sentence) to the extent that they could imagine treating the sentence as non-maximally true. For the negative sentences, such contexts were harder to imagine, hence the low rate of intermediate responses.

### Motivation and provisional conclusion

Our proposal jointly captures both homogeneity and non-maximality, based on three components. First, we associated with sentences containing plural definites a set of candidate propositions. Second, we assumed that only strongly relevant candidate propositions are relevant to the final truth-conditions (this is what accounts for non-maximality). Third, we stated that a sentence is considered true (false) if all these strongly relevant candidate propositions are true (false).

We believe that each of the three components is conceptually quite natural. Regarding the first point, the core idea is that plural predication is intrinsically underdetermined/underspecified, mirroring an intuition prevalent in the previous literature.

The second component can be motivated as follows. When hearing a sentence that has multiple construals, the hearer will eliminate meanings which she knows the speaker could not have intended. Assuming that a cooperative speaker only communicates strongly relevant propositions, the hearer will thus rule out those construals which are not strongly relevant. Saying that the speaker only communicates strongly relevant propositions is just saying that the speaker does not provide superfluous information.

The third component can be thought of as a possible solution to a coordination problem. In case several candidate propositions have passed the test of strong relevance, the hearer is faced with a problem: which of them did the speaker mean? And the speaker is faced with a symmetric problem: how can she be sure that the hearer will pick the proposition she intended? The worst thing that could happen, in a cooperative conversation, would be for the hearer to come to believe something that the speaker believes is false, as a result of the speaker’s utterance. Suppose, then, that a sentence is underspecified between several readings and cannot be fully disambiguated by contextual considerations, which is the case if several candidate propositions pass the test of strong relevance. In order to be on the safe side, the speaker should only use such a multiply ambiguous sentence if *whatever interpretation is selected among those that are ‘reasonable’ given the context*, the interpreter forms a true belief, i.e. only if all the construals that are reasonable in the context are true. The hearer herself can reason in this way, and so the principle of ‘truth on all readings’ can end up being a convention of use between the speaker and the hearer. While such a story should be given a formally explicit form within a decision-theoretic framework (see Spector 2017 for such a proposal), our goal here is only to argue that the principle we are introducing is not only motivated empirically (as we have just argued), but is also conceptually plausible enough that it is worthwhile to pursue a theory that is built on it.^20^

Now, it is clearly not the case that every type of semantic indetermination gives rise to the kind of interpretation strategy we are describing here.^21^ For instance, one might say that a structurally ambiguous sentence like *Someone observed a man with a telescope* is, in some way, semantically underdetermined. Yet it is clear that the sentence is never interpreted to mean that someone observed a man who possessed a telescope by means of a telescope. However, it is plausible enough that ‘truth on all readings’ applies not to surface forms, but to *Logical Forms*, and therefore does not apply to sentences that are structurally, scopally, or lexically ambiguous. The telescope sentence is simply a surface form that can correspond to two different, and individually unambiguous, logical forms.

It is less clear what to say about the case of pronouns. There is a sense in which a sentence like *She arrived* is semantically indeterminate—the pronoun might refer to one of multiple individuals. However, when the reference is unclear, a breakdown of communication ensues. It is not the case that the hearer interprets the sentence as meaning that all relevant female individuals arrived. One might possibly assimilate this to the case of structural ambiguity by reifying referential indices syntactically in a very substantive way; beyond that, all we have to say at this point is that intuitively, the kind of semantic underdetermination that pertains to pronouns is a *different* kind than the one we see with plural predication. What differentiates the particular kind of underdetermination we find with definite plurals, which appears to be some kind of vagueness in a pre-theoretic sense, from other phenomena which one might also want to call so, is a point we have to leave open.

## Comparison with Križ (2016)

In this section, we would like to briefly compare our approach to that of Križ (2016). In our system, sentences are associated with a set of bivalent semantic values, and trivalence as well as non-maximality arise from the way that pragmatics tells us to deal with these sets of propositions. Križ’s system has a different architecture: he assumes that sentences have a fundamentally trivalent semantics, the possibility of non-maximality is the result of the way in which pragmatic principles tell us to use trivalent propositions.

The underlying trivalent semantics is assumed to be virtually identical to ours in terms of overall truth and falsity conditions that arise for sentences with negation, propositional connectives, and quantifiers.^22^ To account for non-maximal readings, Križ proposes to weaken the standard Gricean Maxim of Quality and adapt the Maxim of Relevance to the trivalent setting. His pragmatic assumptions are the following:
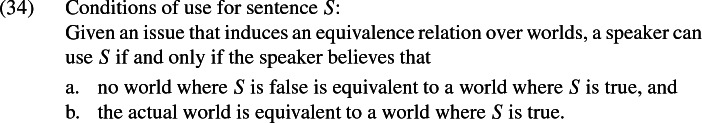
 Condition (34a) enforces the right kind of alignment between the partition and the trivalent proposition expressed by *S*: there must not be a cell in the partition that is compatible with both the truth and the falsity of *S*. This condition is similar in spirit to Strong Relevance, but more general: it has Strong Relevance as a special case for bivalent propositions. The condition (34b), which is a weakened version of the Maxim of Quality, requires the sentence to be, in an intuitive sense, true enough for current purposes, but allows us to ignore deviations from truth that don’t matter, i.e. those that don’t put us in a different cell of the partition.

This approach and ours make subtly different predictions regarding non-maximal readings, two of which we will now discuss (in Sect. 6.1, we point out another case, where Križ’s account might make better predictions, and sketch a potential solution within our framework).

Consider Križ’s example (35) in the context of a job interview where the committee consists of ten professors and the issue at hand is whether or not the interview went well.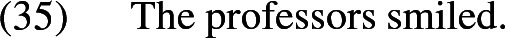 On this approach, the pragmatic interpretation of the sentence is ‘At least one professor smiled, and the interview went as well as if all the professors had smiled’. We will generally infer, then, given general background knowledge, that most of the professors smiled. But as Križ points out, the system also predicts inferences about what the non-smiling professors did instead of smiling, such as that they did not look visibly angry or disappointed. This seems to be a good prediction which is lacking in our system, where nothing follows about the non-smiling professors.

Once we take a closer look, however, this may turn out not to be so much of a problem. Any sentence normally implies that nothing outrageous happened that would undercut the conclusion that it prima facie supports. In this case, the conclusion is that the interview went quite well, and so there is an implication that there was no sign of particular displeasure from any of the professors. That the non-smiling professors didn’t look annoyed or angry is just a special case of this, but so is the inference that no professor, while smiling, made any rude gestures. The latter inference is not predicted by Križ’s theory, because it does not pertain to an exception, so an independent mechanism has to assumed in any case. We are content to rely on this mechanism to derive also inferences about what the exceptions do.

We now turn to a second case of divergent predictions, where our approach can deal more easily with the challenge posed by a particular type of example.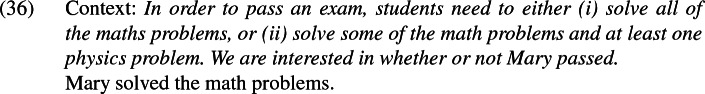 The issue, by stipulation, consists of just two cells reflecting whether Mary passed or not. Since all the worlds where the sentence is false (those where she solved none of the math problems) are in the cell where she failed, and all the worlds where the sentence is true (those where she solved all of the math problems) are in the cell where she passed, condition (34a) is fulfilled. In order for condition (34b) to be met, Mary needs to have passed, and that is predicted, on Križ’s account, to be the overall interpretation of the sentence.

Intuitively, however, such a non-maximal interpretation does not seem to be available. Noticing this problem, Križ speculates that perhaps hearers refuse to intuitively accommodate such a highly specific, but disjunctive current issue, and that once the situation is described to them as above, they would automatically be interested in the manner in which Mary passed the exam. It would then be impossible to obtain intuitive judgements that reflect the non-maximal interpretation that would arise if the issue actually were as described in (36), because it is, in fact, never so. Our approach is in a better position here: instead of mere speculation, we can point to a hard constraint that prevents the sentence from being used to address an issue of this form. Our pragmatic mechanism filters candidate interpretations by strong relevance; but it turns out that in the context given, none of the candidate interpretations of (36) is strongly relevant. The maximal candidate—the proposition that Mary solved all of the math problems—isn’t relevant because it is a proper subset of the cell where she passed. The other candidates are not strongly relevant because they overlap with both cells: given that Mary solved at least some of the math problems, whether she passed depends on whether she solved all of them and on whether she also solved some of the physics problems. The candidate interpretations of (36), however, are silent on this point: they say nothing about the physics problems. Consequently, none of the candidates is strongly relevant, and the sentence cannot be used to address the issue. Given that it has, in fact, been used, the hearer can infer that the issue must be more fine-grained: the speaker must be interested in conveying in which way Mary passed. Then the maximal candidate is relevant, and the interpretation obtained is that Mary passed by solving all the math problems.

This concludes part one of this paper. Clearly, our story is far from complete at this point, as we have not yet specified a concrete mechanism for generating candidate propositions. While this might seem a trivial matter at the outset, it turns out to be quite involved as soon as we consider more complex sentences. Furthermore, we haven’t said anything about how plural expressions interact with quantification, and, specifically, about how the word *all* works (as we have seen, *all* removes both homogeneity and non-maximality). The goal of the next sections is to fill these gaps by providing an explicit recursive semantics that generates the candidates we need, and stating an adequate lexical entry for *all*. We start by establishing a number of desiderata that a theory needs to fulfill (Sect. 5.1), and then offer a system designed to satisfy these (Sect. 5.3).

## Recursive semantics

Having established what the set of candidate interpretations for various sentences with definite plurals should look like, the next step is to look for a compositional or at least recursive semantics that derives these candidate denotations for arbitrary sentences. It turns out to be surprisingly difficult to formally capture the intuitions that we have established in the previous section without unintended side-effects, thus simultaneously fulfilling all the desiderata we are going to set out below.

### Desiderata

The following sections will look in some detail at four desiderata that any formal system for generating appropriate candidate interpretations needs to fulfil.
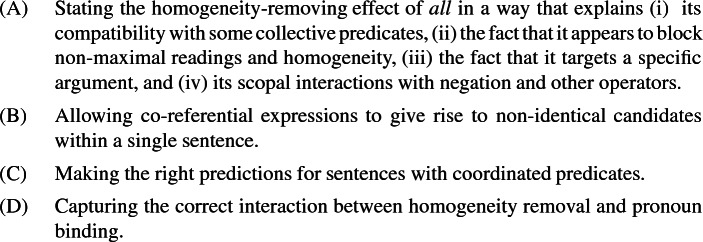


**(A)** ***All*** **as a homogeneity and non-maximality remover, and a quantifier at the same time**

The addition of *all* either in the DP or in adverbial position removes homogeneity and non-maximality with respect to the definite plural DP that it associates with. In some way, the effect of *all* must be that the alternatives that arise from interpreting its associated plural DP are cut down to the universal one.

If *all* were simply a universal quantifier over atoms, every predication would be atomic and homogeneity would be rendered vacuous.^23^ However, this is not a possible solution because of the compatibility of *all* with some collective predicates. That is to say, an analysis for *all* must be able to account for its compatibility with (at least some) collective predicates.

It is furthermore important that *all* removes homogeneity and non-maximality only with respect to one argument, but keeps it with respect to other arguments. (37) still shows homogeneity and non-maximality with respect to the books, so subject-associated *all* must retain the alternatives arising from the definite plural in object position.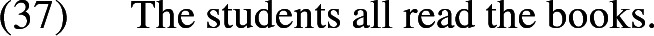 Finally, while *all* removes homogeneity and non-maximality on the DP it associates with, it also appears to scopally interact with negation when the negated predicate is distributive. For instance, (38a) means that not every student smiled, but (38b) (which sounds slightly odd) means that no student smiled.^24^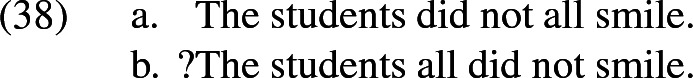 In contrast to (38b), both sentence in (39) are quite natural.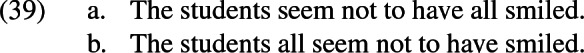 These two sentences obviously do not mean the same thing, which illustrates the more general point that the scope of *all* matters to interpretation. This is of course entirely expected on a standard view in which *all* is a quantifier. Our point here is that *all* cannot be described only as removing homogeneity and non-maximality on the argument it is associated with: the standard, quantificational view also captures something real about the meaning *all*. We need a theory that accounts for both the quantificational nature of *all* and the fact that it removes non-maximality and homogenity.^25^

**(B) Allowing co-referential expressions to give rise to non-identical candidates within a single sentence**

Consider the following sentence: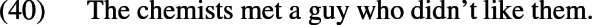 We ignore here the reading in which ‘them’ is morphologically plural but is distributively bound by *the chemists* (this reading would amount to ‘Every chemist met a guy who does not like him’, and would be compatible with every chemist *x* meeting a certain guy who likes all chemists but *x*), and focus on the reading where ‘a guy’ is understood as referring (so to speak) to a specific person that does not co-vary with chemists. Intuitively, it seems possible to interpret ‘the chemists’ as denoting the sum of all chemists, and the pronoun ‘them’ as standing for an existential quantifier over chemists or plurality of chemists. That is, a possible interpretation for (40), and in fact the most salient one, amounts to “all (or nearly all) the chemists met a guy who does not like any of them (or at most likes only a few of them)”. If candidate interpretations only included propositions in which *the chemists * and *them* are interpreted as corresponding to the same candidate, such a reading could not be predicted. Suppose, for simplicity, that candidate denotations are just the universal quantifier and the existential quantifier. If *the chemists* and *them* had to denote the same candidate meaning, we would have only two candidate interpretations for (40), expressed in (41):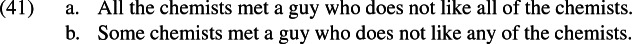 None of these sentences is equivalent to the intended interpretation, and their conjunction is not either.

So we need our system to generate candidate interpretations for sentences in such a way that co-referential expressions are not necessarily interpreted *uniformly* within a single sentence.

**(C) Coordinated predicates**

If the candidates are strictly speaking alternative denotations of the definite plural DP, unwelcome predictions ensue for conjoined and disjoined predicates. For conjunction, we predict that, regardless of non-maximality, the *same* candidate interpretations should fulfill both predicates. (42a), for example, would, informally speaking, give rise to the candidates in (42b). But (42c), which is a possible non-maximal reading of the sentence, is not among these candidates, nor can it be expressed in terms of those with the help of logical connectives.
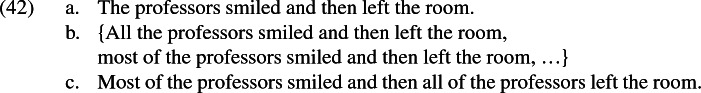


**(D) Homogeneity and binding**

The interaction between homogeneity removal and pronoun binding is simple to state: there is none. That is to say, homogeneity removers affect only the argument position that the expression they are associated with fills. Importantly, *all* does not remove homogeneity for pronouns that are bound by the argument it associates with.

Consider the sentence (43), which has several readings, two of which are informally represented in (43a) and (43b), where $\textsc {dist}$ is a distributivity operator (which introduces universal quantification over atomic parts of a plurality). In (43b), the pronoun is bound by the distributivity operator: every boy believes that he himself will win. The sentence is false if no boy believes that he will win, but he may believe that others will. In (43b), on the other hand, the pronoun is bound directly by the plural DP. On this reading, the sentence is true if every boy believes that every boy will win, and false if no boy believes that any boy will win.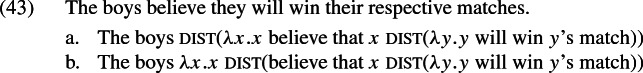 It is this second reading that we are interested in. This kind of reading becomes more accessible when *all* is added in the embedded clause, since it would make no sense to add *all* if the bound pronoun denoted a single individual, as it does in (43a).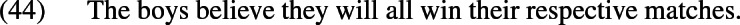 Let us first establish that this is (or can be), indeed, a bound reading and not merely accidental coreference. The first piece of evidence for this is that the predicate “believe they will all win” can be distributed over a conjunction of plurals. (45) is true if all the boys believe that all the boys will win their respective mathes, and all the girls believe that all the girls will win theirs.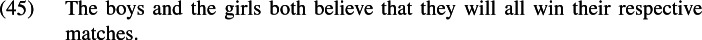 The second piece of evidence (due to Irene Heim, p. c.) is the possibility of sloppy readings with ellipsis. (46) has a reading on which it is true in the same situations as (45).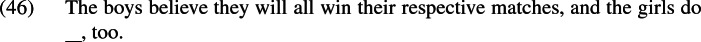 Now let us see what happens to homogeneity and, more visibly, non-maximality depending on where *all* is inserted in (43). Without *all*, homogeneity and non-maximality appear with respect to both the plural DP and the pronoun. The sentence is true if (roughly) all boys believes that (roughly) all boys will win, and false of (roughly) no boys believe that (almost) any of them will win.

If *all* is inserted in the matrix clause, homogeneity and non-maximality disappear with respect to the matrix subject position, but persist in the embedded clause. (47) is true if all the boys believe that (roughly) all the boys will win, and false as soon as one boy believes that (almost) none of them will win.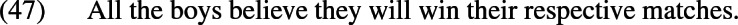 Conversely, inserting *all* in the embedded clause has the opposite effect of removing non-maximality and homogeneity in the embedded clause (we are not replacing *win their respect matches* with just *win*, to make the presentation simpler, but the important point is that we intend a distributive construal of *win*).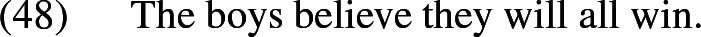 (48) is true if (roughly) all the boys believe that strictly all the boys will win. Note that permitted exceptions from the matrix subject are not, as it were, passed down into the embedded clause. If, in context, we consider it sufficient for nine of ten boys to have the relevant belief, then these nine boys still have to believe that all *ten* boys will win. It is not sufficient for them to believe that the nine of them will win.

This means that simply replacing the definite by its alternative denotations will not do. Consider for instance a case where the subject ‘the boys’ is understood as meaning ‘most boys’. This reading cannot correspond to the LF in (49), where we simply replace ‘the boys’ with ‘more than half the boys’. The reason is that (49) would mean that there is a group *G* which contains more than half of the boys such that each member of *G* believes that every member of *G* will win. But the non-maximal reading we want to capture is one where more than half of the boys believe that *every* boy (not just every member of a subgroup of the boys) will win.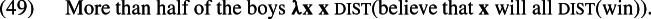 And in the absence of a non-maximal reading, the falsity conditions of (48) take into account homogeneity with respect to the matrix clause only: the sentence is false if (roughly) all boys fail to believe that all boys will win, i.e. if (roughly) all the boys believe that *not all* the boys will win. Again, it is important that the alternatives for (48) not be of the following form, where *Q* is a ‘candidate’ meaning for *the boys*.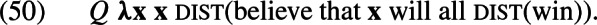 To see why, it will be easier to reflect on the intuitive truth-conditions of the negative counterpart of (48), as in (51):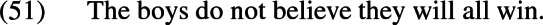 If alternatives for (48) were defined as in (50), (48) would count as false, and (51) as true, if and only if no subset *a* of the boys believed that all of *a* will win. That is, (51) would be expected to imply that no boy (or nearly no boy) believes that he will himself win, which seems much too strong. Rather, (51) is understood to mean that no boy (or nearly no boy) believes that every boy will win (which is compatible with some boys believing that they will themselves win).

In the next sections, we provide an explicit compositional semantics which meets the desiderata we have just described.^26^

### Logical forms

In order to give a semantic theory that fulfills the desiderata just discussed, we find it necessary to assume that the syntactic structures which are the input to semantic interpretation contain information about which argument position of which predicate a quantificational DP fills. This is implemented in the form of *argument indices*, which are different from the usual referential indices that play a role in the interpretation and binding of pronouns.

In particular, a predicate with *n* arguments comes with an *n*-tuple of (sentence-wide) unique argument indices.^27^ We write indices corresponding to structurally higher argument positions first, so that in the case of a transitive verb, the first index corresponds to the subject position and the second index corresponds to the object position.

Quantifiers such as *all* (whether in adverbial or determiner position) carry a set of indices that contains the indices of the argument position(s) that they are associated with. Usually, this set will be a singleton (as in (52c) and (52d)), in which case we ommit curly brackets, but in the case of coordinated verb phrases, it contains different indices from several verbs (exemplified in (52f) and (52h))

We assume that the distributivity operator $\textsc {dist}$, available in the object language, also carries an index in the same way as an intransitive verb, since it takes as its individual argument the plurality that is to be distributed over.
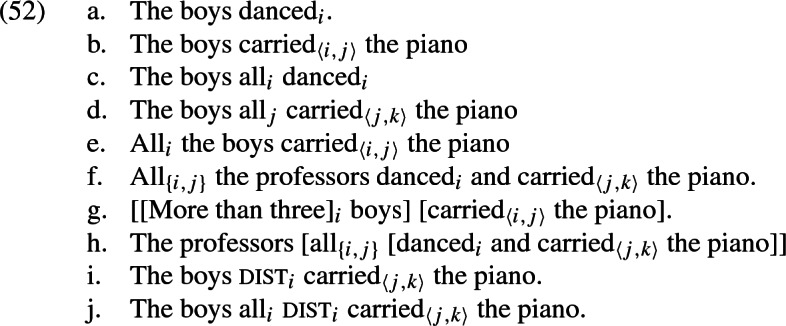
 We will not go into the details of how to define a syntactic theory that yields such structures, but merely note that the kind of information transmission between a predicate and its arguments that we require is far from an outlandish idea and is in essence similar to the notion of agreement.

### Compositional semantics

In this section, we discuss how the logical forms specified above can be interpreted in a (weakly) compositional manner so as to yield sets of candidate interpretations for sentences in accordance with the desiderata we discussed. We follow the assumptions and notational conventions of Heim and Kratzer (1998) unless otherwise noted.

#### The ingredients of plural predication

Our semi-formal discussions so far have assumed that candidates arise from different ways of interpreting a definite plural. However, it has been argued by Löbner (2000) and Križ (2016), partly based on the behaviour of predicate conjunctions, that homogeneity effects are, in fact, rooted in the predicate. What this means for our account is that, intuitively, we are not literally dealing with candidate interpretations of a DP, but rather with candidate interpretations of plural predication: a definite plural just unambiguously denotes a particular plurality, and candidates come into play when that plurality is combined with a predicate.

Our interpretation function $\llbracket \cdot \rrbracket$ is relativised, as usual, to a model, a variable assignment, and a world, but in addition, it also depends on a *homogeneity parameter* $\mathcal {H}$. For a given value of $\mathcal {H}$, the output is one candidate interpretation.

Formally, an admissible homogeneity parameter is a function that takes as its argument an argument index (a number) and an individual and returns a generalised quantifier that is a candidate derived from that individual in the sense of (20). Thus, for all numbers *n* and individuals *x*, $\mathcal {H}(n,x)\in \text {Cand}_x$. It enters into meaning via the interpretation of (homogeneous)^28^ predicates which have argument indices, as well as the distributivity operator. Importantly, in our logical forms, the *nominal restrictors* of determiners do not have any argument index, and the parameter $\mathcal {H}$ does not play any role in their interpretation.^29^ The rules for unary and binary relations are given below, with $\mathcal {I}$ being the model’s interpretation function for lexical constants.^30^
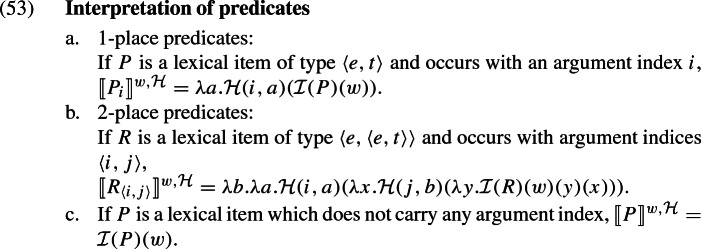
 Thus, for example, we obtain the interpretations in (55).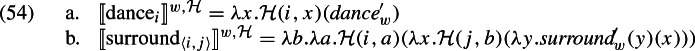 For improved legibility, we will notationally treat candidates as if they were individuals instead of generalised quantifiers and write instead: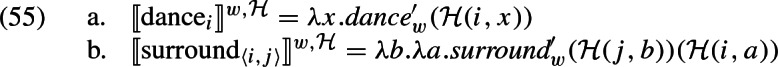 The definite article is interpreted simply as a function that maps a one-place predicate to the maximal individual in its extension (as defined by the relation of mereological parthood).^31^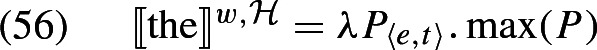 To see how these rules work on a simple case, consider (57) under its non-distributive interpretation.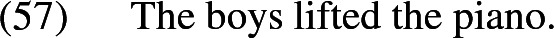 For simplicity, we treat the VP *lift the piano* as if it were a lexical intransitive verb.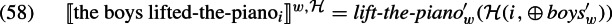 This, of course, is purely extensional, while our candidate interpretations, which need to be assessed for relevance, are propositions, not truth values. They are simply obtained by abstracting over the world variable *w*. Thus, the candidate interpretation generated by a given $\mathcal {H}$ is a proposition as in (59) (omitting now the world parameter on the left-side).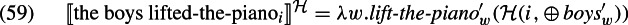 To obtain the whole set of candidate interpretations, we simply plug in all admissible values for $\mathcal {H}$. Assuming that the extension of *boys* is constant across worlds (say, because it is common knowledge who the boys in question are), and that there are just two boys *a* and *b*, the set of candidate interpretations is, informally described, as in (60).
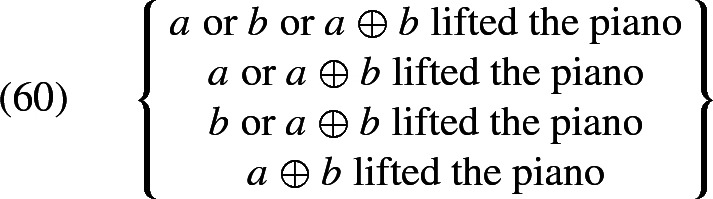
 Turning now to negative sentences, candidate interpretations for a negated plural predication end up equivalent to the negations of the candidate interpretations for the original predication, irrespective of the relative scope of negation and the plural definite. To see this, consider (61), under the two LFs indicated: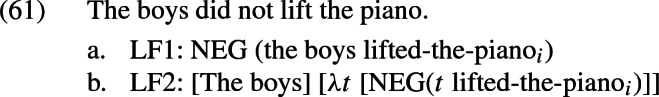 In the case of (61a), it is straightforward to see that the set of resulting candidate meanings consists in the negations of the candidate meanings in (60). Let us look at (61b). We need to introduce assignment functions to interpret the variable *t*. We have:
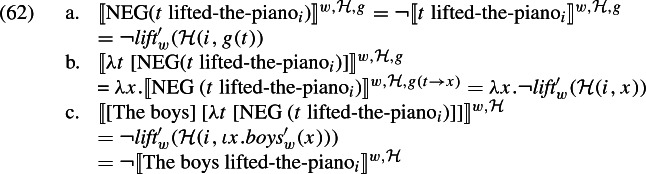
 We also want to capture phrasally distributive readings, such as the reading of (57) on which each boy lifted the piano individually. To serve this purpose, we use a distributivity operator dist, which introduces universal quantification over atomic parts of a plurality. This means that it renders vacuous the homogeneity introduced by the lexical verb (on the relevant argument position that is being distributed over): when *x* is an atom, then $\mathcal {H}(i,x)$ is always just (the Montagovian individual) *x* itself. However, phrasal distributivity is still homogeneous, so we need the distributivity operator to re-introduce candidate formation. This is why dist carries an argument index like a verb and has an index-dependent interpretation. We state its meaning in terms of a meta-language distributivity operator $\Delta$, which abbreviates the standard universal quantification over atomic parts of a plurality.
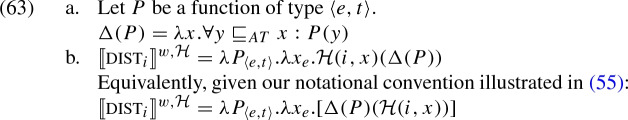
 To see how this plays out, consider the somewhat unrealistic (64), which requires every boy to have lifted a piano by himself.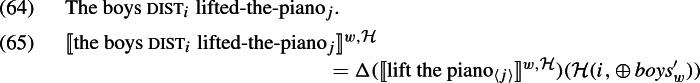 Let us unpack this.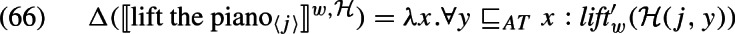 The candidate denotation associated with an atomic individual *y* is always (the Montagovian individual corresponding to) *y* itself. Consequently, the index *j* ends up playing no role in the interpretation and (66) is equivalent to (67).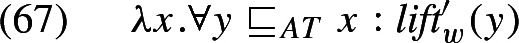 In (65), we are applying to this predicate the generalised quantifier $\mathcal {H}(i,\oplus \,\textit{boys}'_w)$, which is a disjunction $D_1\vee \dots \vee D_n$. (64) is then true if (67) is true of at least one such $D_i$, i.e. if for some $D_i$, every atomic part *x* of $D_i$ is in $\textit{lift}'_w$.

Again, to obtain the actual set of candidate interpretations, we abstract over the world parameter and look at all admissible values for the parameter $\mathcal {H}$. If again the denotation of boys is constant in all worlds and is just ${\{a, b, a\oplus b\}}$, this set will look as in (68) (because entailment relations now exist between disjuncts, which allows us to simplify candidate interpretations):
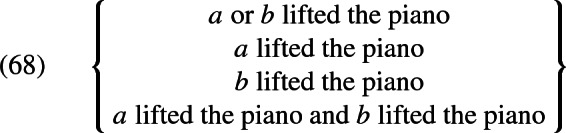
 Going back to negative sentences, once phrasal distributivity is introduced by the dist operator, it is, in principle, possible for negation to take scope either below or above it: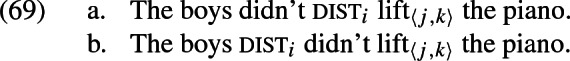 However, this does not make a difference, as the meaning of the distributivity operator is such that it commutes with negation in the following sense: no matter whether negation takes scope above or below dist, the set of candidate interpretations that we obtain in the end is the same.^32^ That is to say, given any candidate interpretation generated by a homogeneity parameter $\mathcal {H}_1$ with low-scope negation, we can find an admissible parameter $\mathcal {H}_2$ that yields the same candidate interpretation when combined with wide-scope negation, and vice versa. Formally,

$$\begin{aligned} \forall \mathcal {H}_1\exists \mathcal {H}_2:\lnot \mathcal {H}_1(i,x)(\Delta (P))=\mathcal {H}_2(i,x)(\Delta (\lambda x.\lnot P(x)))\text { and } \textit{vice}\ \textit{versa}. \end{aligned}$$

^33^

#### Capturing the interactions of *all* (and other quantifiers) with homogeneity and non-maximality

We now turn to the interpretation of *all*, starting with its adverbial (floating quantifier) use (where it combines with a verb phrase) before proceeding to its use as a determiner. We will demonstrate that our proposal meets *Desideratum A* from above and briefly discuss how the relevant features of our analysis play out with other quantificational determiners.

**5.3.2.1 Adverbial** ***all***

Every occurrence of *all* carries an index, and we want *all* to remove homogeneity and non-maximality with respect to the argument position(s) with which its indices are associated. We also want it to be able to enter into scopal interactions with negation and other operators.

We treat *all* as a *universal quantifier over candidate interpretations*; or more formally, as quantifying over (a particular subset of) the possible values of the homogeneity parameter $\mathcal {H}$. Which particular subset this is depends on the indices of *all*: it quantifies over all those admissible parameters that differ from the contextually supplied parameter only with respect to the indices carried by *all*. For example, we will analyse *The girls all lifted the piano* as expressing the conjunction of all the possible candidate interpretations for *The girls lifted the piano*. In the case of *The girls all talked to the boys*, we want to take the conjunction of all the propositions that correspond to all the ways the subject argument of *talked* can be interpreted, keeping constant the interpretation of the object *the boys*—that is, *the boys* will be interpreted, as it would have been, relatively to the contextually supplied parameter, because *all* is co-indexed only with the subject argument.

We first need to define the notion of two parameters being *equivalent except for a set of indices*.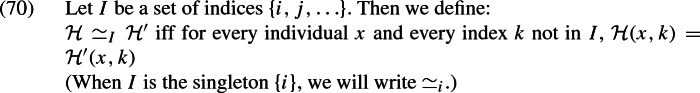 Our semantics for adverbial *all* is then the following: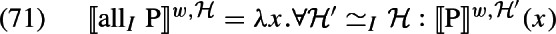 Let us see what results from this for the following sentence:
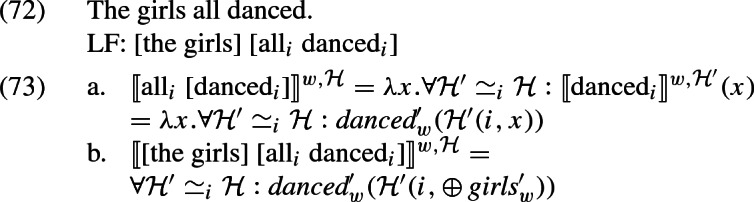
 The proposition we obtain by abstracting over the world variable *w* does not depend on the way the ‘initial’ parameter $\mathcal {H}$ interprets the index *i*, and since *i* is the only index that occurs, the interpretation of the sentence does not depend on $\mathcal {H}$ at all. Consequently, in any given world, it is either true for all choices of $\mathcal {H}$ or false for all choices of $\mathcal {H}$. Since we say that a sentence is undefined in a world if it is true for some choices of $\mathcal {H}$ and false for others, this sentence is never undefined, that is to say, it is bivalent. This is how *all* removes homogeneity.

At the same time, we also capture how *all* removes non-maximality with respect to the argument it is associated with. To see this in a simple example, assume that the extension of *girls* is constant across worlds and consists only of *a* and *b* (and their sum). Then the set of possible $\mathcal {H}'(i,\oplus \,\textit{girls}'_w)$ (generated by chosing different values for $\mathcal {H}'$) is ${\{a\text { or } b\text { or } a\oplus b, a \text { or } a\oplus b, b\text { or } a\oplus b, a\oplus b\}}$. The meaning of (72), given (73), is the conjunction of all the propositions that arise from these candidates, listed in (74a). This conjunction is simply (74b)—the desired result.
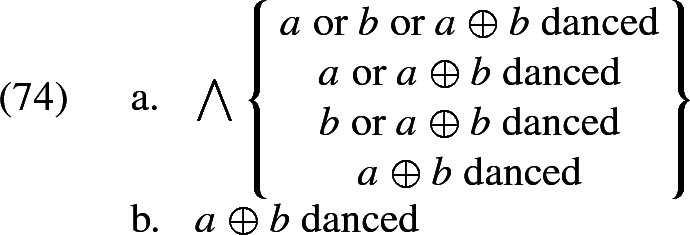
 Since all of this is independent of the choice of the overall parameter $\mathcal {H}$, (74b) is then the only candidate interpretation of (72), and so no non-maximality, which would arise from excluding some candidate interpretations on the basis of relevance, is possible.

Let us now demonstrate how this analysis correctly captures the way in which the relative scope of negation and *all* matters for the overall reading. It is easy enough to see what happens when the whole sentence (73) is embedded under negation, with *all* taking narrow scope:
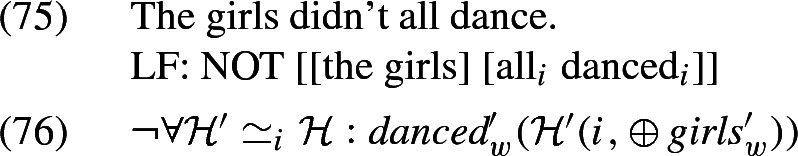
 Here we obtain the candidate interpretations simply by negating the candidate interpretations of the unnegated sentence (the singleton of (74b) above), and so we obtain the singleton of *not(*${a \oplus b}$ *danced)*; that is to say, the sentence is true if and only if either *a* or *b* didn’t dance, and false if both of them did.

Now consider the case where *all* scopes over negation: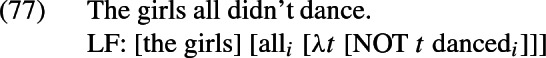 We obtain the following meaning: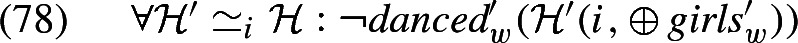 This is true if and only if no girl danced. If any single girl had danced, then we could just choose a $\mathcal {H'}$ such that $\mathcal {H}'(i,\oplus \,\textit{girls}'_w)$ is the candidate based on that girl, which would falsify the universal quantification over parameters.

Another desideratum we noted is that *all* should remove homogeneity and non-maximality *selectively*, i.e. only with respect to the argument(s) it is associated with. This desideratum is satisfied, since *all* quantifies only over homogeneity parameters that are identical on all the indices except those subscripted to *all*. In all our examples above, there was only one plural argument, whose index matches with that of *all*, and as a result we ended up with a unique candidate interpretation, which explained both the unavailability of non-maximal readings and the absence of homogeneity effects. In a case such as (79), however, the presence of a plural object will continue to give rise to several candidate interpretations, and so non-maximality and homogeneity will be preserved for the object argument.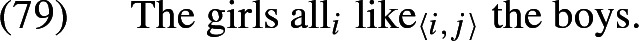 Consider first the version of the sentence without *all*: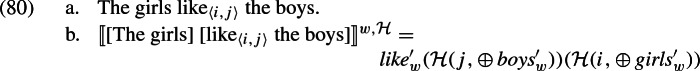 Assuming that in every world compatible with common knowledge there are two relevant girls *a* and *b*, and two relevant boys *e* and *f*, the set of candidate interpretations is sketched in (81).
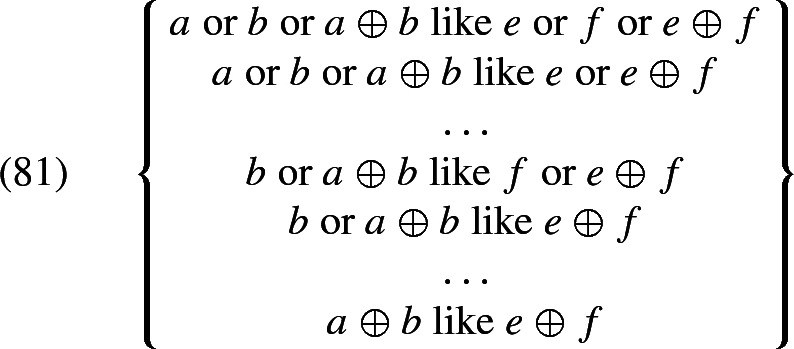


The situations where all of these candidate interpretations are true are those where ${\textit{like}'_w(e\oplus f)(a \oplus b) = 1}$.^34^ The situations where they are all false are those where no girl likes any boy.

The addition of *all* in (79) neutralises the role of $\mathcal {H}$ in the interpretation of the argument it is coindexed with, i.e. the subject. Informally speaking, what happens in this. Each candidate interpretation in (81) corresponds to a certain choice of parameter $\mathcal {H}$. For each such choice, the effect of *all* is that the candidate interpretation generated by $\mathcal {H}$ in (81) is replaced with the conjunction of all the candidate interpretations generated by parameters $\mathcal {H}'$ that agree with $\mathcal {H}$ regarding the object index *j*. That is, each candidate interpretation *m* in (81) is turned into the conjunction of all candidate interpretations $m'$ in (81) that agree with *m* on the object side. Due to entailment relations between candidate interpretations, this will just amount to a proposition of the form ${a \oplus b}$ *like* *x*, where *x* is a certain candidate denotation for the object (the one chosen by $\mathcal {H}$). That is, the resulting set of candidate interpretations is reduced compared to (81), but is not a singleton, and looks as follows:
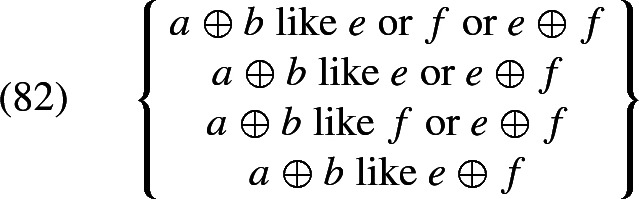
 In a context where every piece of information is relevant, the sentence will be judged clearly true if all candidate interpretations are true, i.e. if ${a \oplus b}$ like ${e\oplus f}$. In this case, therefore, the resulting pragmatic truth-conditions are identical to that of the sentence without *all*. Once we look at contexts that would allow for a non-maximal understanding, however, we find a difference: no non-maximal reading for the subject is predicted to be possible for (79) even in contexts that would support such a non-maximal reading for its counterpart without *all*, because the candidate interpretations do not vary with respect to the subject, and so excluding candidate interpretations as irrelevant will not change anything in this respect.

Furthermore, even in a context where every piece of information is relevant, (79) is predicted to have, as it were, a larger range of falsity conditions than (80): it is false as soon as one of the girls fails to like any boy, as that immediately makes all the candidate interpretations false (whereas (80) is only false when no girl likes any boy). Note that the sentence with *all* is still not fully bivalent: if both *a* and *b* like *e*, but do not like *f*, then some candidate interpretations are true and others are false, so that the sentence is overall undefined.

**5.3.2.2 Determiner** ***all***

Consider the following sentence: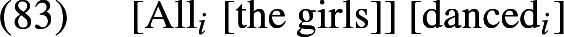 We want this to be equivalent to *The girls all danced*. More generally, as far as we can see, all the facts we have discussed and will discuss in the next sections regarding the interactions of adverbial *all* with non-maximality, homogeneity and variable binding remain identical when we move to determiner *all*. Within our framework, we need a syncategorematic rule to capture all these facts.
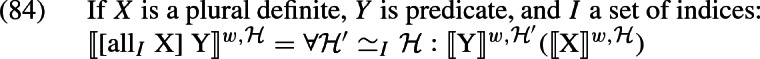


**5.3.2.3 Other quantifiers**

On a standard semantics for quantifiers such as, say, *more than three*, the current system overgenerates non-maximal readings and homogeneity effects. Let us illustrate why this is so before we show how our approach to *all* can be extended to other quantifiers.

Consider the following standard lexical entry for *more than eight*: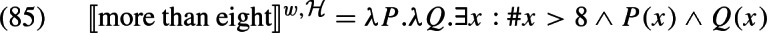 Then we have: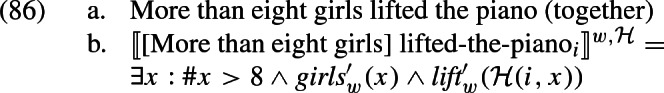 Let $\mathcal {H}$ be the parameter that maps every pair (*i*, *x*) to a quantifier equivalent to ‘all of *x* except maybe one or two’. Then the resulting interpretation will be equivalent to *More than six girls lifted the piano*. Clearly, such a ‘non-maximal’ reading of the sentence does not exist.

Another aspect of the same problem arises with the negation of (86a).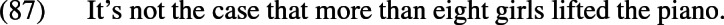 The predicted meaning, relative to a given parameter $\mathcal {H}$, is the following: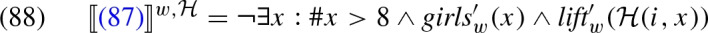 The *strongest* possible candidate interpretation for this is obtained by making $\mathcal {H}$ choose the *weakest* possible candidate interpretations for every individual *x* that occurs as an argument of *lifted-the-piano*$_i$. That is, for every *x*, $\mathcal {H}$ should map (*i*, *x*) to the quantifier equivalent to *at least one atomic member of* *x*. In a context where every candidate interpretation is relevant, the predicted pragmatic interpretation of (88) is the conjunction of all candidate interpretations, which, in this case, just amounts to this strongest possible meaning. This interpretation can be paraphrased as follows: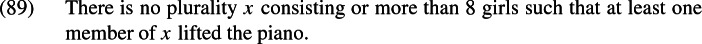 This, in turn, is equivalent to (90), which is, quite obviously, not the empirically observed meaning of (87).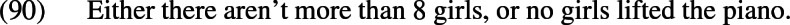 Our solution will consist in making sure that quantifiers such as *more than eight* neutralise the effect of $\mathcal {H}$ with respect to the argument positions they are associated with, just as we did with *all*. Effectively, the meaning of the quantifier *more than eight* is such that it first applies adverbial *all* to its scope. So our official semantics for *more than eight* is given by the following syncategorematic entry: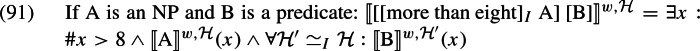 A nice side-effect of this treatment is that it makes floating *all* vacuous in a sentence like (92), which explains why such a sentence sounds infelicitous:
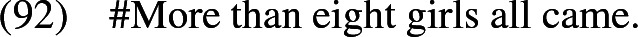


#### A note on restrictors and restrictive relative clauses

In our system, nominal restrictors are not affected by the homogeneity parameter.^35^ The reason for this choice is the following. If *dancers* in *the dancers* could be interpreted non-maximally, then it could be true of a plurality that contains both dancers and non-dancers. As a result, *the dancers* could denote, relative to a certain homogeneity parameter, say, the plurality of all people (some of whom are dancers). Now, in the sentence *The dancers smiled*, the predicate *smiled* can itself be interpreted non-maximally, with the result that the sentence could end up being true, relative to certain homogeneity parameter, in a situation where no dancer smiled.^36^ By making sure that nominal restrictors are not affected by homogeneity parameters, our system avoids this undesirable possibility.^37^

However, even within our system, the problem resurfaces when we consider restrictors that include a restrictive relative clause, as in (93):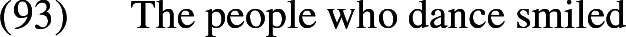 Assume the following LF for (93):^38^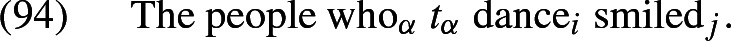 Because both *dance* and *smiled*, relative to some homogeneity parameters, could be interpreted non-maximally, (94) could end up being true if no person who dances smiled. In this case, stipulating that verbs within a relative clause do not have argument indices would not be a reasonable move. Not only is there no plausible syntactic reason why indices on verbs would disappear in relative clauses, it it also the case that non-maximal interpretations can arise in relative clauses. In (95), the NP *the books* can be interpreted non-maximally: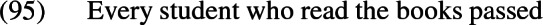 That is, we want to remove non-maximality only on the argument that is relativised. An obvious way to do this consists in making sure that relative pronouns inherit a homogeneity index corresponding to the argument that is being relativised, and assigning them a semantics which, similarly to what we have done above for quantifiers, removes non-maximality and homogeneity on the argument they are coindexed with (the relative pronouns is notated *Op* in (96)):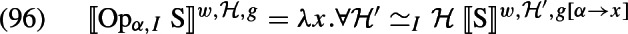 Such a treatment also provides an explanation why, except in very specific contexts that we discuss in the next paragraph, floating *all* is infelicitous in a subject relative clause, as in (97). Floating *all* indeed turns out to be vacuous. This is similar to the explanation we gave for the unacceptability of (92).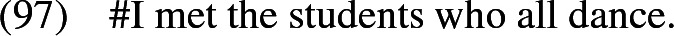 Now, the interpretation of the restrictor of plural definites does in fact raise additional challenges, which may make this treatment too simplistic. Imagine a context where there are several salient groups of students, and in only one of these groups are the vast majority of people dancers. Then it seems that *The dancers* could refer to that group, and this corresponds to a non-maximal reading for *dancers*. Consider now a context where there are several groups of students, all of which include dancers, and only one of these groups is made up entirely of dancers. Then it seems that *The students who all dance* could be used to refer specifically to the group that only includes dancers. But, as just discussed, according to our semantics in (97), *all* is vacuous when it associates with the relativised argument of a relative clause. It seems, though, that for such readings to arise, there needs to be a contextually natural division of students into subgroups—a contextual cover in the sense of Schwarzschild (1996). To capture such effects, we would need to supplement our proposal with an additional context-sensitive component (such as contextual covers). We leave this issue for further research.

#### Coreferential expressions

Co-referential expressions, being associated with different argument positions, and therefore indices, can be interpreted by $\mathcal {H}$ independently of each other. Consider again (40), repeated below as (98):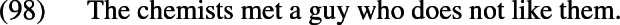 The reading we want to be able to capture is the following: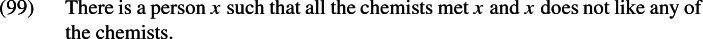 Consider the following parse, in which we omit distributivity operators (we can assume the ‘lexical cumulativity’ hypothesis to dispense with distributivity operators in the case of *meet* and *like*, but nothing crucial hinges on this):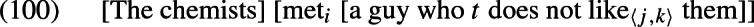 Under this parse, the strongest possible candidate interpretation is exactly the meaning given in (99). This meaning arises if $\mathcal {H}$ maps the subject’s denotation and its corresponding argument index *i* to the quantifier corresponding to the set of all chemists, and the pronoun’s denotation and its corresponding argument index *j* to the quantifier *at least one chemist*. Since the argument indices are different, this is perfectly possible despite the fact that the subject DP and the pronoun denote the same plurality. Furthermore, this candidate interpretation entails all the other candidate interpretations, and so ends up being the pragmatic interpretation of (40) (in a context where the partition representing what is relevant is maximally fine-grained).

#### Coordinated predicates

In a sentence such as (101), we want to allow for the possibility that the candidate denotations of the subject relevant to each of the coordinated predicate are different, even though there is only a single occurrence of the plural definite expression. That is, we want to allow for the possibility that (101), discussed by Križ (2016), has a non-maximal reading relative to *smile* but not relative to *left*—in some contexts, one might for instance understand it at conveying something like ‘Most of the professors smiled and then all the professors left’.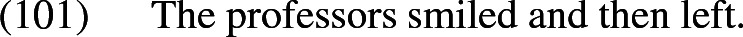 This follows quite directly on our account, since the predicted meaning for (102a) is given in (102b):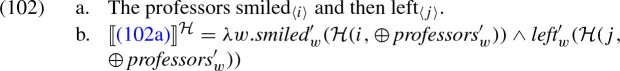 The fact that $\mathcal {H}$ is passed different indices in both conjuncts means that it does not necessarily choose the same candidate disjunctions in both of them, and so causes the set of candidate interpretations to contain something like ‘Most of the professors smiled and then all of the professors left’.

Finally, note that *all* can attach to coordinated predicates or appear as a determiner of the subject of coordinated predicates, as in (103):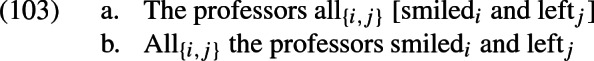 This is the reason why we need *all* to carry a set of indices instead of single indices, since it removes homogeneity with respect to the subject argument of both verbs simultaneously: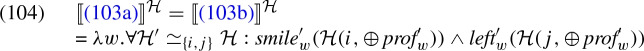 In words: for all the ways in which $\mathcal {H}$ can interpret the argument positions of *smile* and *left*, ‘The professors smiled and left’. This is equivalent to ‘Every professor smiled and left’, as desired.

#### *All*, binding, and coreference

Recall Desideratum (D) from above, where we named the three sentences in (105) as another stumbling block for potential analyses. On the reading of interest, the pronoun *they* is not bound by a distributivity operator, but directly by the subject DP *the boys*, so that the sentences are true only if every boy believes that all the boys (and not only he himself) will win their respective matches.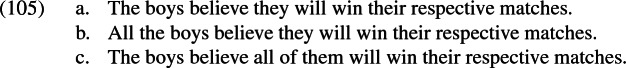 To avoid the need to introduce modals and intensional operators, we will discuss sentences without them that share the same relevant properties: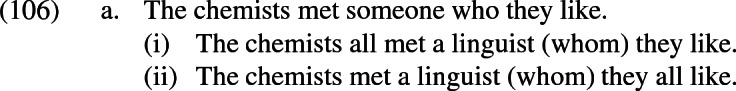 Our system can deal with (106a-i), where homogeneity needs to be removed with respect to the binder without also being removed with respect to the bound pronoun, because the argument indices that govern homogeneity are not the same thing as binding indices. Representing binding indices with Greek letters, its LF is as in (107) on the relevant parse where the matrix subject directly binds the pronoun.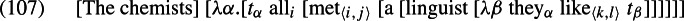 Semi-formally, because *all* carries the index associated with the argument position filled by $t_\alpha$, it collapses, as it were, the candidate interpretations of $t_\alpha$ from $\mathcal {H}(i,g(\alpha ))$ to simple $g(\alpha )$. However, it does not carry the argument index associated with the position of $they _\alpha$, and so the pronouns candidate interpretations remain dependent on $\mathcal {H}$. Hence we obtain the following:
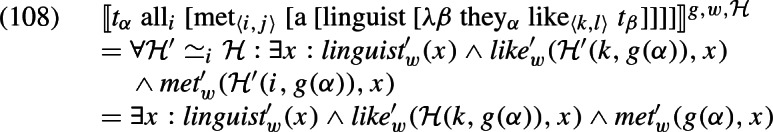
 Binding then simply inserts $\oplus \,\textit{chemists}'_w$ for $g(\alpha )$.

(106a-i) is correctly accounted for by our system because the candidate denotations associated with the binding definite plural are not literally alternative denotations for the DP, but are introduced by the lexical predicate *believe* and are unable to bind anything. Its LF, again on the relevant parse, is (109).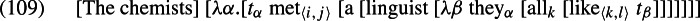 Here, the subconstituent ‘[a [linguist [$\lambda \beta$ they$_\alpha$ [all$_k$ [like$_{\langle k,l\rangle }$ $t_\beta$]]]]]’ gets interpreted as *a linguist that every member of* $g(\alpha )$ *likes* due to the presence of *all*. The larger ‘$[t_\alpha$ met$_{\langle i,j\rangle }$ [a [linguist [$\lambda \beta$ they$_\alpha$ [all$_k$ like$_{\langle k,l\rangle }]]]]]$ is then interpreted as $\mathcal {H}(g(\alpha ), i)$ *met a linguist every member of* $g(\alpha )$ *likes* due to the nature of *meet* as a homogeneity-introducing lexical predicate. Finally, binding from above by *the chemists* again simply leads to the substitution of $g(\alpha )$ with $\oplus \,\textit{chemists}'_w$, so that we obtain $\mathcal {H}(i,\oplus \textit{(}'_wchemists))$ *met a linguist that every member of* $\oplus \, ( chemists)$ *likes*, i.e. *The chemists (potentially non-maximally) met a linguist that every chemist likes*. This is the desired outcome: importantly, the non-maximal interpretation of the matrix subject has no effect on the interpretation of the pronoun it binds.

## Open problems

We would like to conclude by briefly mentioning two open problems and hint at potential solutions. The first one we call *functional non-maximality*, and the second one pertains to the relation between partitions (for the purposes of our mechanisms) and overt questions.

### Functional non-maximality

Suppose that two students have slightly different reading obligations, because, say, they take the same class but do not major in the same disciplines, and so the criteria for passing are more lenient for one of them. For concreteness, suppose that Mary had to read all of the ten books on the reading list, and John had just to read at least eight of them. Then consider the following:
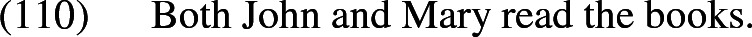


Our impression is that in the specified context, the sentence may be interpreted as conveying that John read at least eight of the books and Mary read all of them. This is in fact exactly what Križ (2016) predicts. On his account, (110) conveys that both John and Mary read at least some of the books, and that the answer to the question whether they satisfied their reading obligations is the same as it would be if both had read all the books—which simply means that the sentence is interpreted as conveying that both of them satisfied their reading obligation. On our account, however, this reading cannot be generated. In fact, the only relevant candidate interpretation in this case is the proposition that both read all of the books.

In order to solve this problem, we think that we could borrow tools that have been used to account for functional readings of definite and indefinite phrases (as in *Both John and Mary like the local bar*, which can mean that John likes the bar that is local relative to his own location, and Mary the one relative to hers). We would need to assume even more complex logical forms, and to make homogeneity parameters take addition arguments. We do not offer such an extension here.

### Overt questions

A reviewer points out that in the following discourse, the natural understanding of B’s answer is (close to) universal: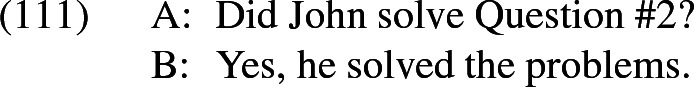 We find this discourse mildly infelicitous for likely unrelated reasons having to do with the (lack of) contrast between *Question #2* and the plain definite *the problems*.^39^ However, we do share the intuition that something more ought to be said, and that our theory’s *prima facie* prediction that, to the extent that it is felicitous, B’s answer should be interpreted existentially, is undesirable. While we do not have a fully worked-out resolution of the problem, the following are some considerations which we believe may point in the right direction.

As already pointed out by Križ (2016), there is almost always uncertainty about what exactly matters in a conversation, and as much as the partition (“that which matters for the purposes of the conversation”) is used to interpret the sentence, so the sentence uttered is itself used to infer what the partition is. A sentence with a definite plural feels ill-suited (intuitively; we do not state here a formal principle that codifies this ill-suitedness) to addressing a question about a single individual (or in general a specific subset of the relevant plurality), so it triggers a search for an additional partition to be resolved, and the resulting uncertainty leads to a somewhat vaguer meaning—in this case, pertaining to the mentioned problems in general. That is, the partition against which such a sentence is interpreted cannot always be pinned down with an overtly asked question. Spector (2017) offers a model of the interpretation of plural definites within the Rational Speech Act framework (very much in the spirit of our proposal in this paper) where listeners perform joint inference about the intended meaning and the intended ‘Question Under Discussion’ (QUD), and where certain answers to questions are interpreted as answering a question which is not the one explicitly asked. The specific proposal in Spector (2017) does not, however, provide a solution to this problem, since this proposal in fact aims to reproduce the qualitative predictions of our approach and that of Križ (2016) within a game-theoretic framework. In the case at hand, the general idea would be that, if one wants to answer a question such as (111), using a less underspecified sentence such as ‘Yes, he did solve Question #2’ would be in some sense better, yielding the inference that the author of (111) probably intended to address a different, more general issue. That is, competition between possible alternative utterances would yield inferences about the intended QUD.

A similar issue arises in cases discussed by Malamud (cf. Sect. 2.2.1), where no explicit question is asked, but the context makes it clear that even one window left open would lead to a disaster in the event of a storm. Despite this fact being established, the interpretation of the utterance *Oh my! The windows are open!* in such a context still seems rather vaguer than and not exactly equivalent to ‘At least one window is open’. At this point, all explicit formal semantic theories of the context-sensitivity of definite plurals face this problem because they rely on relativising their meaning, in some way or other, to issues modelled as discrete partitions. Arguably, progress on this point would necessitate a move to a suitable probabilistic model of pragmatics where the pragmatic interpretation of a given utterance can leave some uncertainty about both the intended truth-conditions of a sentence as well as the underlying issue being addressed, which does not exist yet.

## Conclusion

In this paper, we have presented a theory of the interpretation of plural predication in natural language. Our theory is in the tradition of Krifka (1996) and Malamud (2012), where a sentence with a plural predication is underspecified between several meanings, and pragmatics governs how this underspecified meaning is made precise in context. While keeping this general architecture, however, our theory crucially differs from its predecessors in two respects, which allows us to avoid several shortcomings of previous theories.

First, there is the question of which meanings a sentence with a plural predication, such as (112), is underspecified between. Among these meanings are (112a) and (112b), but we argue that the set of possible interpretations is actually wider than hitherto assumed, and can be obtained by replacing the definite plural with generalised quantifiers of a certain specific form (essentially, all disjunctions consisting of books and pluralities of books; cf. Sect. 3.1).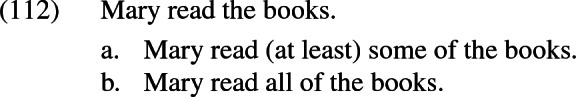 Second, while previous instantiations of this general approach have assumed that *one* of the potential meanings is picked by pragmatics to be the overall meaning of the sentence, according to considerations of logical strength and/or relevance, we argued that candidate meanings are filtered through a relevance constraint and the overall meaning of the sentence is the conjunction of all the meanings that remain. Thus, it is possible for the sentence to have a pragmatic meaning in context that is not identical to any of the candidate meanings. While it may, at first glance, appear strange that a sentence that is underspecified between several meanings should be able to end up meaning something yet different, we argued that there is, in fact, a very natural motivation for our interpretation procedure in terms of communicative strategies (Sect.  3.5).

This theory is underpinned by a recursive semantics that generates the right set of candidate interpretations for complex sentences with negation, coordination, and quantification. In Sect. 5, we first set out a number of desiderata that any such system must meet, and which it turned out to be surprisingly difficult to fulfil simultaneously, before specifying a system that does so.

## Appendix

### An implementation of upward homogeneity

Here we discuss how to modify the definition of candidate denotations to capture the phenomenon of upward homogeneity. In our current definition, the candidate denotations for a plurality *X* are obtained as follows. First you pick a set of parts of *X*, $\{x_1, \ldots , x_n\}$, then for each such $x_i$ you build the disjunction of all the *y*’s that are contained in *X* and that contain $x_i$, and then you take the grand disjunction of everything that is obtained in this way. In order to account for upward homogeneity, we need to allow for candidate denotations which include as disjuncts individuals which are proper superpluralities of *X*. Specifically, the new definition of candidate denotations is the following: Now we define:

$$\begin{aligned} \text {Cand}_x {:}{=}\{\bigvee \text {Conv}_\sqsubseteq (A\cup B)|A\subseteq \mathcal {P}art(x)\wedge B\subseteq {\mathcal Sup}(x)\} \end{aligned}$$

This is a straightforward generalisation of our previous definition in (21), which would be obtained from the above by setting *B* to be constant as $\{x\}$.

In words, we obtain a candidate by performing the following steps:

1. Pick a set *A* of (possibly improper) parts of *x*.
2. Pick a set *B* of individuals which (possibly improperly) mereologically contain *x*.
3. Form the union of *A* and *B*.
4. Form the convex closure of that union. Call that set *S*. Note that *S* necessarily contains *x*.
5. Take the existential quantifier with domain *S*.

Let us see how this new definition of candidate denotations helps us capture upward homogeneity on top of standard homogeneity. To keep the exposition simple enough, we’ll use a predicate that can be true of a small plurality, in fact even of an atom (which is not the case of *surrounded the castle*), as in (114): Suppose there are two girls *a* and *b*, and one boy *c*. The candidate meanings for (114a), based on (113), are then as follows, where the strongest candidate meaning is in boldface and the weakest one is underlined: In a context where everything is relevant, (114a) counts as true if all the candidate meanings in (115) are true. Since the boldfaced candidate meaning entails all the others (it is part of every disjunction), the sentence comes out true just in case the two girls built the raft together, as before. It will count as false if all the candidate meanings in (115) are false, which will be the case if and only if the underlined candidate is false (since it is the weakest candidate). That is, (114a) is false, and (114b) (with negation) is true, if and only if neither of the two girls was part of a plurality that built the raft—this is the desired meaning, which includes the upward homogeneity effect.

We now need to adjust our semantics for *all*. We want *all* to remove standard homogeneity but not upward homogeneity. Consider indeed the following: While this sentence is true if, say, just half of the soldiers formed a circle together around the castle (without anybody else), we don’t want to count it as true if all the soldiers of my brigade together with some other soldiers formed a circle around the castle. Rather, (116) seems to mean that one cannot find a group that contains all the soldiers of my bridage and who formed a circle around the castle. So we don’t want *all* to universally quantify over all candidate interpretations, but only to a subset of those. The following definitions for *all* and auxiliary notions in terms of which the meaning of *all* is defined, does the work. The critical move consists in modifying the equivalence relation over homogeneity parameters in terms of which *all* is defined. To see how this works in a concrete case, consider again the toy example above, with two girls and one boy, relative to the following sentences: The candidate denotations for *the girls* are the following (cf. (115)): For the first five of them, the maximal individual witness is ${a \oplus b}$, and for the last five, it is ${a \oplus b \oplus c}$. Now, what *all* does is the following, informally: for each possible choice of a candidate denotation (i.e. all possible homogeneity parameters), it, so to speak, replaces it with the conjunction of all candidate denotations that share the same maximal individual witness. Because of the entailment relationships between candidate meanings, we thus end up with the following two candidate meanings for (120a): Now the sentence is true if both candidate meanings are true, i.e. if the first candidate is true (since it entails the other one), i.e. if the two girls built the raft together and without collaborating with anyone. But for it to be false, it has to be that the second candidate is false, i.e. that there is no plurality containing the two girls such that this plurality built the raft. As a result, the negative sentence in (120b) ends up meaning that it is not the case that both girls *participated* in building the raft. Thanks to *all*, we lose ‘standard homogeneity’, and (120b) is true if just one of the two girls built the raft on her own, or took part in building the raft, but it is false not only if the two girls built the raft on their own, but also if they both took part in building the raft with some other people.

## Footnotes

1 Note that Brisson discusses only non-maximality, but does not link it to homogeneity in any way (nor does her approach to non-maximality lend itself to such a connection). Burnett does not discuss homogeneity, but her trivalent approach allows for a connection between non-maximality and homogeneity. For a detailed discussion of those works, see the appendix of Križ (2016), which we endorse. For comments on Lasersohn, see fn. 25. Let us note that none of these works, including Križ (2016), provides a fully compositional account of *all* (Križ 2015, however, does).

2 Lasersohn (1999) and Brisson (1998) discuss non-maximality but not homogeneity.

3 Examples (32a) and (32b), respectively, in Krifka (1996).

4 This notion of truth/falsity as “truth/falsity on all admissible readings” is clearly closely related to the intuitions behind supervaluationism and Strong Kleene logic. Our implementation differs from both, though the patterns we predict for quantificational sentences are the same as on Strong Kleene logic (though our system is more general).

5 $\sqsubseteq$ is the relation of mereological parthood and ${\oplus }$ the mereological fusion operation. We assume a standard mereological account of pluralities, cf. Link (1983) or Landman (1991). Here, as in the following, we are sloppy about distinguishing between expressions (definite descriptions, sentences, etc.) and their denotations (individuals, propositions, etc.). For a more formally rigorous reconstruction of Malamud’s theory in the same spirit, see Križ (2016).

6 In the real world, non-maximal readings arise more easily with higher numbers of objects and are difficult to obtain when only very few are concerned. However, choosing such a low number for illustration allows us to write out all the alternatives.

7 Things would be different if we assumed that homogeneity holds in the meta-language. Given homogeneity, the candidate interpretation represented by “Mary didn’t read ${a \oplus b \oplus c}$” would be the proposition that Mary didn’t read any of the three books, and would then count as the only maximally relevant candidate. But this would amount to stipulating homogeneity instead of explaining it.

8 Judgments might be clearer for a sentence such as *Only one girl read the books.*

9 Arguably, in some contexts, collective predicates do display such participatory readings, perhaps through some sort of coercion, but we take it to be clear that this is not the case here, especially with the addition of *together*.

10 For a more detailed empirical discussion of homogeneity and non-maximality with collective predicates, we refer to Križ (2016).

11 Thus, in our terminology, candidate *denotations* are, loosely speaking, possible ways of interpreting the definite description, and so they are generalised quantifiers over individuals. Candidate *interpretations* are ways of interpreting the whole sentence, and so they are propositions.

12 Or rather, since we are talking about quantifiers, the Montagovian individual based on *x*, which is $\lambda P.P(x)$. In the following, we will freely identify individuals and Montagovian individuals for ease of notation. Hence, we will speak of the disjunction of two individuals when what we mean is the generalised quantifier that is the disjunction of the corresponding Montagovian individuals.

13 We thank an anonymous reviewer for suggesting this formulation in terms of existential quantifiers over a convex domain.

14 See the appendix for a further extension of this based on a slight change in the set that is used as the basis for forming the convex closure.

15 See Bar-Lev (2018, chapt. 4) for a different approach to homogeneity with non-distributive predicates, which for reasons of space we cannot discuss or evaluate here.

16 To be sure, undefinedness was diagnosed in other types of scenarios, such as (i).

17 If no candidate proposition is strongly relevant, these two universal quantificational statements (‘all the strongly relevant propositions are true/false’ ) are both trivially true, and so the sentence is predicted to be judged both true and false. This looks problematic, but we may plausibly assume that in order for the sentence to be felicitous, at least one candidate denotation has to be strongly relevant.

18 Križ’s *homogeneity constraint* is phrased in terms of the trivalent meaning of predicates directly; his system does not involve anything like candidate interpretations. The constraint forbids a predicate to be simultaneously false of *a* and true of *b* when *a* and *b* overlap:

19 To see the relevant scoping relationship, keep in mind that in (33) the plural definite ‘the windows’ is interpreted as being restricted to a domain which is itself bound by the quantifier, i.e. these sentences are merely a more natural rendering of:

20 We have provided a pragmatic motivation for truth being characterised as ‘truth on all readings’, but not for falsity being identified to ‘falsity on all readings’. This second component follows if we assume that the falsity-conditions of a sentence are the truth-conditions of its negation.

21 Thanks to two anonymous reviewers for raising this point, as well as to a number of colleagues who made the same point when we presented this work.

22 Križ (2016) does not present a compositional account, but an explicit formal system is supplemented in Križ (2015, chapt. 2).

23 Cf. fn. 32 for a treatment of floating *each* along these lines.

24 Replacing *smile* with a collective predicate leads to even further degradation (unless maybe the subjects is understood to quantify over groups of students, and the whole sentence ends up meaning that for every group *x* of students, the members of *x* did not collectively lift the piano):  We do not account here for the relative degradedness of (38b) or (i).

25 Lasersohn (1999) proposes a theory in which sentences with plural definites are associated with a set of interpretations that constitutes the ‘pragmatic halo’ of the sentence, and treats *all* as contracting this set to the unique maximal interpretation. However, this approach does not allow for an adequate compositional treatment of negation, *all*, and their scopal interaction.

26 Even though we only deal with a small part of English, we think that the ideas we present here can be extended to more complex constructions if needed. We chose to simply present a system which makes reasonable predictions without motivating every aspect of it and comparing it to other potential systems—suffice it to say that we investigated a number of possible choices and found the one we are presenting to be the most empirically adequate one.

27 In the case of intransitive verbs, we omit the angled brackets that indicate a tuple.

28 This leaves open the possibility of non-homogeneous lexical predicates. Such predicates do, indeed, exist, but Križ (2015) argues that they are scarce and constrained to predicates that have, in some way, to do with measuring a quantity, such as *numerous*. See Bar-Lev (2018, chapt. 4) for a different take on such non-homogeneous predicates.

29 See Sect. 5.3.3 for further discussion.

30 In the following, we will write $\textit{dance}'_w$ for $\mathcal {I}(\text {dance})(w)$, etc.

31 This function is, of course, partial, reflected in the presuppositionality of singular definite descriptions. We ignore this fact here. All our examples involve plural nouns, whose denotation we assume to be closed under mereological fusion, such that it is always the case that $\max (P)=\oplus P$. We will occasionally employ the latter notation for readability.

32 Importantly, DIST should not be viewed as a silent version of floating *each*. Floating *each* scopally interacts with negation and removes homogeneity. A straightforward semantics for *each* consists in treating it as an object-language version of our meta-language operator $\Delta$.  So we have:  The homogeneity parameter $\mathcal {H}$, when its second argument *y* is atomic, simply returns *y*, and is therefore vacuous in such a case. As a result, *each danced* has the same denotation with respect to every homogeneity parameter, so homogeneity is removed, and *each* scopally interacts with other operators in the usual way.

33 Before providing a general proof, let us illustrate with an example. Suppose that ${\mathcal {H}_1(i,a \oplus b \oplus c) = a \vee (b \oplus c) \vee (a \oplus b) \vee (a \oplus b \oplus c)}$ (which is a well-formed candidate denotation). Given the definition of $\Delta$, $\mathcal {H}_1(i,x)(\Delta (P))$ reduces to $P(a) \vee (P(b) \wedge P(c))$. Hence, by de Morgan’s laws, ${\lnot \mathcal {H}_1(i,a \oplus b \oplus c)(\Delta (P))}$ can be written as ${\lnot P(a) \wedge (\lnot P(b) \vee \lnot P(c))}$, which is itself equivalent to $(\lnot P(a) \wedge \lnot P(b)) \vee (\lnot P(a) \wedge \lnot P(c))$. Let $\mathcal {H}_2$ be such that ${\mathcal {H}_2(i, a \oplus b \oplus c) = (a \oplus b) \vee (a \oplus c) \vee (a \oplus b \oplus c)}$ (which is also a well-formed candidate denotation for ${a \oplus b \oplus c}$). We now have: ${\lnot \mathcal {H}_1(i, a \oplus b \oplus c)(\Delta (P)) = \mathcal {H}_2(i,a \oplus b \oplus c)(\Delta (\lambda x. \lnot P(x)))}$. The general proof works in the same spirit. Proof: Assume $\mathcal {H}_1(i,x)$ denotes a certain disjunction $\bigvee \text {Conv}_\sqsubseteq (\{x,y_1,\dots ,y_n\})$. Given the definition of $\Delta$, this disjunction can be reduced to its minimal elements in $\mathcal {H}_1(i,x)(\Delta (P))$: $\mathcal {H}_1(i,x)(\Delta (P)) = (y_1 \vee \ldots \vee ~y_n)(\Delta (P))$. Now, each $y_i$ is a sum of plural individuals, $a_{i,1} \oplus \ldots \oplus a_{i,n}$, and thanks to the definition of $\Delta$, we have $\Delta (P)(y_i) = P(a_{1}) \wedge \ldots \wedge P(a_{n,k})$. So $\mathcal {H}_1(i,x)(\Delta (P)$ can be expressed as: $(P(a_{1}) \wedge \ldots \wedge P(a_{n})) \vee \ldots \vee (P(z_{1}) \wedge \ldots \wedge P(z_{m}))$.xxx Given de Morgan’s laws, we have $\lnot \mathcal {H}_1(i,x)(\Delta (P) = (\lnot P(a_{1}) \vee \ldots \vee \lnot P(a_{n})) \wedge \ldots \wedge (\lnot P(z_{1}) \vee \ldots \vee \lnot P(z_{m}))$. This is a *conjunctive* normal form expression based on atomic propositions of the form $\lnot P(x)$, and it can itself be rewritten as a *disjunctive* normal form based on the very same atomic propositions, i.e. something of the form $(\lnot P(\alpha _{1}) \wedge \ldots \wedge \lnot P(\alpha _{k})) \vee \ldots \vee (\lnot P(\omega _1) \wedge \ldots \wedge \lnot P(\omega _l))$ (by repeated application of the identity $(p \vee q) \wedge r = (p \wedge r) \vee (q \wedge r)$). Let us then consider the GQ *G* defined as $\bigvee \text {Conv}_\sqsubseteq (\{\alpha _1 \oplus \ldots \oplus \alpha _k, \ldots , \omega _1 \oplus \ldots \oplus \omega _l \})$. By construction, we have $\lnot \mathcal {H}_1(i,x)(\Delta (P)) = G(\lnot \Delta (P))$. Since *G* is a candidate denotation for *x*, there is a parameter $\mathcal {H}_2$ such that $\mathcal {H}_2(i,x) = G$. In other words, for every parameter $\mathcal {H}_1$, there is a parameter $\mathcal {H}_2$ such that $\lnot \mathcal {H}_1(i,x)(\Delta (P))=\mathcal {H}_2(i,x)(\Delta (\lambda x.\lnot P(x)))$. The proof for the *vice versa* direction works on the same principles.

34 We do not unpack at this point what $\textit{like}'_w(y)(x)$ actually means, that is to say, which situations make it true, and in particular whether lexical cumulativity (Champollion 2010 and others) obtains. All we need to assume is that $\textit{like}'_w(y)(x)$ is not true if at least one part of *y* likes no part of *x* or at least one part of *x* is not liked by any part of *y*, which seems to us to be indisputable.

35 Meanwhile, definite plurals that occur as arguments inside a restrictor, such as in relative clauses, are affected as normal. Along with all existing theories, we predict a preference for an existential interpretation of plural definites in all downward-entailing contexts. It is not clear that this is desirable for downward-entailing restrictors and antecedents of conditionals. For instance, an existential interpretation for the plural definites is not very natural in a sentence such as *Every student who solved the problems passed*. We think something similar takes place with gradable adjectives. While trivalent approaches to vagueness predict that *Every tall student came* should convey that every student who is clearly tall or borderline tall came, such a sentence seems to be rather interpreted as meaning that every student who is *definitely* tall passed. We leave the issue of restrictors and conditionals for further research.

36 We thank an anonymous reviewer and Roger Schwarzschild for raising this issue.

37 This means that adjectives modifying nouns in restrictors should also occur without any index.

38 We use Greek letters for ‘binding’ indices on pronouns, traces, or any expression that receives a value from the assignment function.

39 Compare the much more natural:

## References

[CR1] Bar-Lev, Moshe E. (2018). *Free choice, homogeneity, and innocent inclusion*. Hebrew University of Jerusalem dissertation.

[CR2] Brisson, C. (1998). *Distributivity, maximality, and floating quantifiers*. Rutgers Univerity dissertation.

[CR3] Burnett H (2012). From quantification and intensification to SLACK regulation. Proceedings from the Annual Meeting of the Chicago Linguistic Society.

[CR4] Champollion, L. (2010). *Parts of a whole: Distributivity as a bridge between aspect and measurement*. University of Pennsylvania dissertation.

[CR5] Chierchia, G., Fox, D., & Spector, B. (2012). Scalar implicatures as a grammatical phenomenon. In C. Maienborn, K. von Heusinger, & P. Portner (Eds.), *Semantics: An international handbook of natural language meaning* (Vol. 3, pp. 2297–2331). Berlin: Mouton de Gruyter.

[CR6] Dalrymple M, Kanazawa M, Kim Y, Mchombo S, Peters S (1998). Reciprocal expressions and the concept of reciprocity. Linguistics and Philosophy.

[CR7] Dalrymple, M., Kanazawa, M., Mchombo, S., & Peters, S. (1994). What do reciprocals mean? In M. Harvey, & L. Santelmann (Eds.), *Proceedings of SALT IV* (pp. 61–78). Ithaca, NY: Cornell University.

[CR8] Fox D, Spector B (2018). Economy and embedded exhaustification. Natural Language Semantics.

[CR9] Heim I, Kratzer A (1998). Semantics in generative grammar.

[CR10] Krifka, M. (1996). Pragmatic strengthening in donkey sentences and plural predications. In T. Galloway, & J. Spence (Eds.), *Proceedings of SALT VI* (pp. 136–153). Ithaca, NY: Cornell University.

[CR11] Križ, Manuel (2015). *Aspects of homogeneity in the semantics of natural language*. University of Vienna dissertation.

[CR12] Križ M (2016). Homogeneity, non-maximality, and ‘all’. Journal of Semantics.

[CR13] Križ M, Emmanuel C (2015). Two methods to find truth-value gaps and their application to the projection problem of homogeneity. Natural Language Semantics.

[CR14] Landman F (1991). Structures for semantics.

[CR15] Lasersohn P (1999). Pragmatic halos. Language.

[CR16] Link, G. (1983). The logical analysis of plurals and mass terms: A lattice-theoretical approach. In R. Baeuerle, C. Schwarze, & A. von Stechow (Eds.), *Meaning, use and interpretation of language*. New York: de Gruyter.

[CR17] Löbner S (2000). Polarity in natural language: Predication, quantification and negation in particular and characterizing sentences. Linguistics and Philosophy.

[CR18] Magri, G. (2014). An account for the homogeneity effects triggered by plural definites and conjunction based on double strengthening. In S. Pistoia Reda (Ed.), *Pragmatics, semantics and the case of scalar implicatures* (pp. 99–145). Houndsmills: Palgrave Macmillan.

[CR19] Malamud S (2012). The meaning of plural definites: A decision-theoretic approach. Semantics & Pragmatics.

[CR20] van Rooij R (2003). Questioning to resolve decision problems. Linguistics and Philosophy.

[CR21] Sabato S, Winter Y (2012). Relational domains and the interpretation of reciprocals. Linguistics and Philosophy.

[CR22] Schwarzschild, R.(1996). *Pluralities*. Dordrecht: Kluwer Academic. Publishers.

[CR23] Spector, B. (2013). Homogeneity and plurals: From the strongest meaning hypothesis to supervaluations. Presented at Sinn und Bedeutung 18. https://ehutb.ehu.eus/uploads/material/Video/3289/Sinn18_01.pdf.

[CR24] Spector, B. (2017). The pragmatics of plural predication: Homogeneity and non-maximality within the rational speech act model. In Cremers, A., van Gessel, T., & Roelofsen, F. (Eds.), *Proceedings of the 21st Amsterdam colloquium* (pp. 435–444). Amsterdam. https://semanticsarchive.net/Archive/jZiM2FhZ/AC2017-Proceedings.pdf.

[CR25] Winter Y (2001). Flexibility principles in Boolean semantics.

